# Influence of the intestinal microbiota on the immunogenicity of oral rotavirus vaccine given to infants in south India

**DOI:** 10.1016/j.vaccine.2017.11.031

**Published:** 2018-01-04

**Authors:** Edward P.K. Parker, Ira Praharaj, Anna Zekavati, Robin P. Lazarus, Sidhartha Giri, Darwin J. Operario, Jie Liu, Eric Houpt, Miren Iturriza-Gómara, Beate Kampmann, Jacob John, Gagandeep Kang, Nicholas C. Grassly

**Affiliations:** a Department of Infectious Disease Epidemiology, St Mary’s Campus, Imperial College London, London, UK; b Division of Gastrointestinal Sciences, Christian Medical College, Vellore, India; c Imperial BRC Genomics Facility, Commonwealth Building, Hammersmith Hospital, London, UK; d Division of Infectious Diseases and International Health, University of Virginia, Charlottesville, VA, USA; e Centre for Global Vaccine Research, Institute of Infection and Global Health, University of Liverpool, Liverpool, UK; f NIHR Health Protection Research Unit in Gastrointestinal Infections, University of Liverpool, Liverpool, UK; g Department of Paediatrics, St Mary’s Campus, Imperial College London, London, UK; h MRC Unit The Gambia, Fajara, Gambia

**Keywords:** Enteropathogens, Immunogenicity, Microbiota, Rotavirus, Rotarix

## Abstract

Oral rotavirus vaccines have consistently proven to be less immunogenic among infants in developing countries. Discrepancies in the intestinal microbiota, including a greater burden of enteropathogens and an altered commensal community composition, may contribute to this trend by inhibiting the replication of vaccine viruses. To test this possibility, we performed a nested case–control study in Vellore, India, in which we compared the intestinal microbiota of infants who responded serologically or not after two doses of Rotarix delivered at 6 and 10 weeks of age as part of a clinical trial (CTRI/2012/05/002677). The prevalence of 40 bacterial, viral, and eukaryotic pathogen targets was assessed in pre-vaccination stool samples from 325 infants using singleplex real-time PCR on a Taqman array card (TAC). In a subset of 170 infants, we assessed bacterial microbiota composition by sequencing the 16S rRNA gene V4 region. Contrary to expectations, responders were more likely than non-responders to harbor ≥1 bacterial enteropathogen at dose 1 (26% [40/156] vs 13% [21/157] of infants with TAC results who completed the study per protocol; χ^2^, *P* = .006), although this was not apparent at dose 2 (24% [38/158] vs 23% [36/158]; *P* = .790). Rotavirus shedding after dose 1 was negatively correlated with the replication of co-administered oral poliovirus vaccine (OPV). We observed no consistent differences in composition or diversity of the 16S bacterial microbiota according to serological response, although rotavirus shedding was associated with slightly more bacterial taxa pre-vaccination. Overall, our findings demonstrate an inhibitory effect of co-administered OPV on the first dose of Rotarix, consistent with previous studies, but in the context of OPV co-administration we did not find a strong association between other components of the intestinal microbiota at the time of vaccination and Rotarix immunogenicity.

## 1 Introduction

Each year, an estimated 215,000 children die of severe gastroenteritis associated with rotavirus infection, including between 47,000 and 79,000 in India [1], [2]. Although two internationally-licensed oral rotavirus vaccines, Rotarix (RV1) and RotaTeq, are currently available, their efficacy is impaired in low-income countries [3]. Mechanisms responsible for this phenomenon remain uncertain, but may include maternal antibodies, histo blood group antigen phenotype, malnutrition, environmental enteropathy, and interference by enteric infections [4], [5], [6], [7]. In a systematic review of oral poliovirus vaccine (OPV) trials, we observed a reduction in the odds of seroconversion and vaccine virus shedding among individuals infected with non-polio enteroviruses (NPEVs) [8]. Similarly, during a recent study in Bangladesh, enterovirus quantity at the time of immunization was negatively correlated with the immunogenicity of both OPV and RV1 [9].

The composition of the bacterial microbiota may also shape response to oral vaccines. Viruses exploit microbiota-derived compounds to replicate efficiently in the intestinal mucosa, as evidenced by the reduced pathogenicity of poliovirus and rotavirus in antibiotic-treated mice [10], [11]. Significant geographic variation occurs in the composition of the infant microbiota [12], [13], which may in turn contribute to discrepancies in vaccine performance.

We carried out a nested case–control study among infants enrolled in a clinical trial of RV1 immunogenicity in India [14]. Herein, we tested the hypothesis that failure to seroconvert would be associated with an elevated pathogen burden and an altered bacterial microbiota composition.

## 2 Materials and Methods

### 2.1 Study population

Full details of the study design, laboratory procedures, and statistical analyses are provided in the Supplementary Methods. Samples were obtained from a randomized, placebo-controlled trial assessing the impact of daily supplements of zinc and/or probiotics (*Lactobacillus rhamnosus* GG) on the immunogenicity of RV1 and OPV doses co-administered at 6 and 10 weeks of age [14]. The trial was performed in Chinnallapuram, a densely populated urban area in Vellore, India [15]. Infants were considered eligible for enrollment if they were between 35 and 41 days of age, weighed at least 3.2 kg, were available for the duration of the follow-up period, and had no medical conditions that precluded involvement. Written informed consent was obtained from parents or guardians prior to recruitment. Infants received routine vaccines according to the national schedule in India, including OPV at birth, but were excluded if they had received any other doses of OPV or rotavirus vaccine.

Serum anti-rotavirus VP6 IgA antibodies were measured at 6 and 14 weeks of age using an antibody-sandwich enzyme immunoassay [16]. Rotavirus seroconversion was defined as a four-fold increase in anti-VP6 IgA concentration or detection of antibodies at ≥20 U/ml in previously seronegative infants. Hereafter, we refer to infants who seroconverted to rotavirus as responders and infants who failed to seroconvert as non-responders.

Following completion of the trial, we conducted a nested case–control study to assess the association between enteropathogens and RV1 response. Infants were considered eligible for the study if they received supplements or placebo, received scheduled doses of OPV and RV1, and provided paired serum samples. To meet sample size requirements (Supplementary Methods), we analyzed stool samples from all responders, subject to constraints in sample availability (n = 162). We randomly selected an approximately equal number of non-responders from each study arm (n = 163) to account for the potential confounding of treatment group with enteropathogen burden. Baseline characteristics were comparable between responders and non-responders (Table 1).

**Table 1:** Baseline characteristics.

	Enteropathogen subset			Microbiota subset		
	Responders (n = 162)	Non-responders (n = 163)	*P*	Responders (n = 85)	Non-responders (n = 85)	*P*
Completed the study per protocol	159 (98.1)	161 (98.8)	1.000	85 (100)	84 (98.8)	1.000

Treatment group						
Placebo	36 (22.2)	37 (22.7)	-	32 (37.6)	31 (36.5)	-
Zinc	35 (21.6)	34 (20.9)		0 (0.0)	0 (0.0)	
Probiotics	39 (24.1)	39 (23.9)		34 (41.2)	35 (40.0)	
Zinc/probiotics	52 (32.1)	53 (32.5)		19 (22.4)	19 (22.4)	
Age at enrollment (days)	35.8 (1.8)	35.9 (1.9)	0.577	36.0 (1.8)	36.0 (2.0)	0.933
Female	86 (53.1)	88 (54.0)	0.912	42 (49.4)	46 (54.1)	0.645

Mother’s education						
None	11 (6.8)	8 (4.9)	0.901	4 (4.7)	4 (4.7)	0.677
Primary	24 (14.8)	29 (17.8)		10 (11.8)	13 (15.3)	
Secondary	86 (53.1)	87 (53.4)		50 (58.8)	42 (49.4)	
Higher secondary	25 (15.4)	25 (15.3)		12 (14.1)	18 (21.2)	
Degree/diploma	16 (9.9)	14 (8.6)		9 (10.6)	8 (9.4)	

House type						
Kutcha (temporary materials)	10 (6.2)	9 (5.5)	0.255	7 (8.2)	3 (3.5)	0.240
Mixed	55 (34)	70 (42.9)		28 (32.9)	36 (42.4)	
Pucca (permanent materials)	97 (59.9)	84 (51.5)		50 (58.8)	46 (54.1)	

Health status						
Any breastfeeding at enrollment	162 (100)	162 (99.4)	1.000	85 (100)	85 (100)	1.000
Positive for rotavirus IgA at baseline	42 (25.9)	45 (27.6)	0.802	18 (21.2)	26 (30.6)	0.220
Diarrhea at 6 or 10 weeks	11 (6.8)	12 (7.4)	1.000	5 (5.9)	7 (8.2)	0.766
Stunted at 6 or 10 weeks	37 (22.8)	51 (31.3)	0.105	22 (25.9)	25 (29.4)	0.732
Underweight at 6 or 10 weeks	25 (15.4)	39 (23.9)	0.069	17 (20.0)	15 (17.6)	0.845

Data are mean (standard deviation) or n (%). Responders and non-responders were compared using Wilcoxon’s rank sum test or Fisher’s exact test. Stunting was defined as a height-for-age *Z* score of <−2 and underweight as a weight-for-age *Z* score of <−2. One non-responder in the microbiota subset was excluded from the final analyses owing to a clerical error that led to inclusion of the incorrect samples.

In a subset of 170 infants that had been assessed for enteropathogen burden (including 85 responders), we sequenced the 16S rRNA gene V4 region in stool samples collected before each RV1 dose to assess the intestinal bacterial microbiota. For this microbiota subset we preferentially sampled recipients of placebo-only and probiotics-only, enabling us to assess the effect of probiotics on microbiota composition as a secondary objective.

### 2.2 Enteropathogen testing by TaqMan array card

Stool samples were obtained on the day of or preceding each vaccine dose. These were kept at room temperature until collection (which typically occurred within 4 h), transported in cold boxes to the laboratory, then stored at −70 °C until testing, with up to two intervening freeze–thaw cycles for aliquoting. We extracted DNA and RNA from 200 mg of the 6- and 10-week pre-vaccination stools from each infant and assessed the presence of 40 enteropathogen targets via real-time reverse transcription PCR (RT-PCR) using TaqMan array cards (TACs) [17], [18]. A threshold cycle (Ct) value of 35 was used as a cut-off for pathogen detection [17]. Enterovirus-positive samples were assessed for the presence of Sabin polioviruses using multiplex RT-PCR [19]. To assess RV1 replication (or ‘take’), we quantified rotavirus shedding in samples collected pre-vaccination (indicative of natural rotavirus exposure) and 4 and 7 days after the 6-week dose using a VP6-specific real-time RT-PCR assay [20], [21].

### 2.3 Characterization of the intestinal microbiota by 16S rRNA gene sequencing

Our laboratory and bioinformatic pipelines for assessment of the bacterial microbiota have previously been described [22]. We amplified the 16S rRNA gene V4 region using primers 515F (5′-GTGCCAGCAGCCGCGGTAA-3′) and 806R (5′-GGACTACCAGGGTATCTAAT-3′) in DNA extracted from stool samples collected at 6 and 10 weeks of age in each infant. Purified PCR products were sequenced in two Illumina MiSeq runs (2 × 151 bp) [23]. Reads were assembled using FLASH [24] and analyzed using QIIME (MacQIIME version 1.8.0) [25]. After quality filtering [26] and chimera removal, sequences were clustered *de novo* into operational taxonomic units (OTUs) with ≥97% nucleotide identity using uclust and taxonomically assigned using the RDP classifier [27].

### 2.4 Statistical analysis

#### 2.4.1 Enteropathogen burden

Analyses were performed on infants who completed the study per protocol (as defined by Lazarus et al. [14]). Our primary outcome was the association between rotavirus seroconversion and the presence of ≥1 enteropathogen at 6 or 10 weeks of age, as determined via logistic regression. We excluded enteroaggregative *Escherichia coli* (EAEC) from the primary outcome analysis based on the high prevalence of this target in an interim analysis and its limited association with diarrhea during previous studies in resource-poor settings using TACs [28], and enteroviruses given that they may reflect replication of OPV rather than natural enteropathogen exposure.

As secondary outcomes, we compared the prevalence of individual pathogens, pathogen groups (bacterial, viral, eukaryotic, or any), mixed infections (defined as >1 enteropathogen), Sabin viruses, and concurrent diarrhea (defined as ≥3 loose stools in a 24-h period within the 7 days preceding vaccination) according to RV1 outcome (seroconversion/shedding) at each dose using the χ^2^ test or Fisher’s exact test (the latter applied if there were <5 infected or uninfected individuals in a given comparison). The presence of an enterovirus in the absence of any Sabin viruses was defined as an NPEV, though notably our assays did not allow distinction of samples positive for both Sabin viruses and NPEVs. For prevalence estimates, 95% confidence intervals (CIs) were calculated using the Clopper–Pearson exact method [29]. Wilcoxon’s rank sum test was used to compare total pathogen count and Ct values at each dose according to RV1 outcome; lower Ct values correspond to higher target copy numbers and were used as an indicator of increased pathogen abundance. Rotaviruses were excluded from analyses of mixed infections, pathogen groups, and pathogen count given that, in contrast to the hypothesized inhibitory effect of enteropathogens, one would expect natural rotavirus exposure or RV1 shedding to be positively correlated with rotavirus seroconversion. Across the 6- and 10-week doses, we assessed the association between the number of doses in which ≥1 enteropathogen was present (0, 1, or 2) and rotavirus seroconversion via logistic regression.

Type 3 poliovirus seroconversion rate was compared according to rotavirus seroconversion status using the χ^2^ test, as was the prevalence of dose 1 rotavirus shedding. To assess the potential impact of poliovirus replication following the birth dose of OPV on the take of OPV administered at 6 weeks, we compared the shedding of enteroviruses (including Sabin serotypes and NPEVs) at 10 weeks of age (i.e., 4 weeks after vaccination) according to whether ≥1 Sabin serotype was present in the 6-week samples (also using the χ^2^ test).

*P* values of .05 were considered significant. For comparisons of prevalence or abundance for individual TAC targets present in at least 1% of the study population, *P* values were adjusted via Benjamini–Hochberg false discovery rate (FDR) correction [30]. All analyses were carried out in the programming language R [31].

#### 2.4.2 Microbiota composition

After quality filtering, we obtained a minimum of 3726 sequences per sample, which we standardized to 3500 sequences per sample. For comparisons of within-sample (alpha) diversity, we evaluated OTU count (overall and within the enteropathogen-rich phylum Proteobacteria) and Shannon index as continuous dependent variables via linear regression. Unweighted and weighted Unifrac distances were used to assess divergence between samples (beta diversity), and cluster significance determined using the adonis function in the R package *vegan* [32]. We also used Unifrac distances between samples collected from the same infant over time as a measure of microbiota stability, and compared this measure between infants using Wilcoxon’s rank sum test. Differences in relative taxon abundance were assessed using a non-parametric test based on a bootstrapped *t* statistic [33]. We report on any associations with a *P* value of <.15 after FDR correction. Random Forest models were fit to discriminate infants according to RV1 outcome and study arm based on OTU abundances [34].

#### 2.4.3 Sensitivity analyses

We carried out sensitivity analyses to assess the influence of Ct threshold, study arm, amplification efficiency of MS2 (the extrinsic RNA control in the TACs), baseline rotavirus-specific IgA status, and seroconversion criteria on the comparisons described above.

## 3 Results

### 3.1 RV1 immunogenicity

A companion paper describes the primary outcomes of the trial [14]. Briefly, out of 551 individuals who completed the study per protocol, 173 (31%) seroconverted to rotavirus, including 54/137 (39%) recipients of zinc and probiotics, 42/136 (31%) probiotics recipients, 40/143 (28%) zinc recipients, and 37/135 (27%) placebo recipients. Infants receiving probiotics (arms 1 + 2) or zinc supplementation (arms 1 + 3) did not differ significantly in their rate of seroconversion compared with placebo recipients (arms 3 + 4 or 2 + 4, respectively). However, a significant increase in seroconversion rate was observed among infants who received both supplements compared with those who received neither (Fisher’s exact test, *P* = .040).

### 3.2 Association between pathogen burden and seroconversion

#### 3.2.1 Primary outcome

We assessed the presence of enteropathogens using TACs in 325 infants (Table 1). Among per-protocol infants (n = 320), we obtained eligible TAC assays (positive for the extrinsic DNA control and at least one RNA target) for 6-week samples in 313 infants, 10-week samples in 316 infants, and 6- and 10-week samples in 309 infants. We observed ≥1 enteropathogen (excluding EAEC, enterovirus, and rotavirus) at either 6 or 10 weeks in 70/154 (45%) non-responders and 78/155 (50%) responders (odds ratio [OR] 1.22, 95% CI 0.78–1.90).

#### 3.2.2 Secondary outcomes

**Fig. 1:**
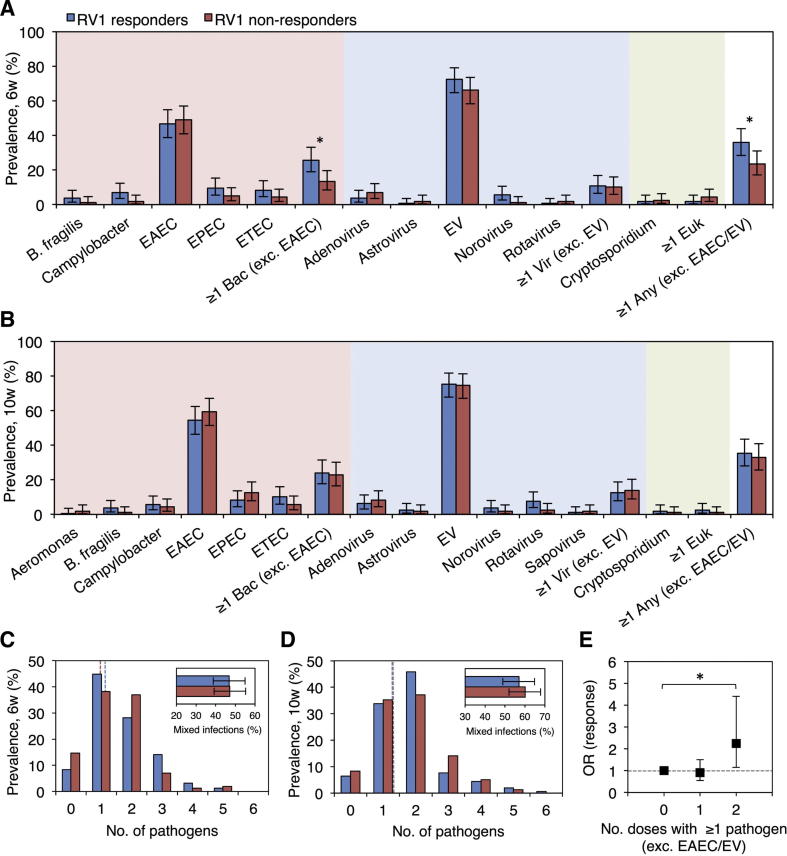
Association between concurrent pathogens and seroconversion after two doses of Rotarix. Prevalence of concurrent pathogens at (A) 6 weeks and (B) 10 weeks of age by seroconversion status. Pathogens present in at least 1% of the study population are included. (C, D) Pathogen count and mixed infection prevalence at (C) 6 weeks and (D) 10 weeks of age by seroconversion status. Mean pathogen counts are indicated by dotted lines. (E) Impact of concurrent enteropathogens at 6 and 10 weeks of age on the odds of seroconversion. Rotaviruses were excluded from analyses of pathogen groups, mixed infections, and pathogen count. ^*^P < .05. Abbreviations: Bac, bacteria; EAEC, enteroaggregative *Escherichia coli*; EPEC, enteropathogenic *E. coli*; ETEC, enterotoxigenic *E. coli*; EV, enterovirus; Euk, eukaryote; OR, odds ratio; Vir, virus; w, weeks.

EAEC and enteroviruses were the predominant TAC targets at 6 and 10 weeks (Fig. 1A and 1B). Their prevalence did not differ significantly according to seroconversion status (Supplementary Table 1), although enterovirus abundance was greater among responders than non-responders at 6 weeks (Ct, 29.5 ± 4.5 [mean ± standard deviation (SD)] vs 31.0 ± 4.1; Wilcoxon’s rank sum, FDR-corrected *P* = .042). The majority of enterovirus-positive samples (155/217 [71%] and 176/235 [75%] at 6 and 10 weeks, respectively) contained ≥1 Sabin serotype (Supplementary Fig. 1).

Other enteropathogens were generally more common in RV1 responders than non-responders at 6 weeks (Fig. 1A), although no individual comparisons of prevalence or abundance were significant after FDR adjustment (Supplementary Table 1). Combining across TAC targets, ≥1 enteropathogen (excluding EAEC, enterovirus, and rotavirus) was observed in 56/156 (36%) responders and 37/157 (24%) non-responders at 6 weeks (χ^2^, *P* = .017). This discrepancy can be attributed primarily to bacterial pathogens, which were more common in RV1 responders than non-responders (40/156 [26%] vs 21/157 [13%] excluding EAEC; χ^2^, *P* = .006). These differences were no longer apparent at 10 weeks (Fig. 1B). The prevalence of viral enteropathogens other than enteroviruses and of eukaryotic enteropathogens did not differ significantly between responders and non-responders at either dose.

We detected up to six pathogens per sample with an average of 1.6 (SD 1.0) and 1.8 (SD 1.0) at 6 and 10 weeks, respectively. The prevalence of mixed infections did not differ significantly according to seroconversion status at 6 or 10 weeks (χ^2^, *P* values >.05), nor did total pathogen count (Wilcoxon’s rank sum, *P* values >.05; Fig. 1C and 1D). Concurrent diarrhea was documented in <5% of individuals at 6 and 10 weeks; this proportion did not differ between responders and non-responders at either dose (χ^2^, *P* values >.05).

#### 3.2.1 Pathogen prevalence over successive doses

Compared with infants clear of enteropathogens at both 6 and 10 weeks, we observed a significant increase in the odds of seroconversion when ≥1 pathogen (excluding EAEC, enterovirus, and rotavirus) was present at both doses (OR 2.25, 95% CI 1.15–4.41; Fig. 1E) – an effect that was absent among individuals infected at only one dose (OR 0.91, 95% CI 0.55–1.50). A similar trend was apparent when considering only bacterial pathogens (OR 1.32, 95% CI 0.76–2.31 and OR 1.98, 95% CI 0.93–4.23 in association with the presence of ≥1 bacterial pathogen [excluding EAEC] at one or both doses, respectively).

### 3.3 Association between enteropathogen burden and RV1 take

**Fig. 2:**
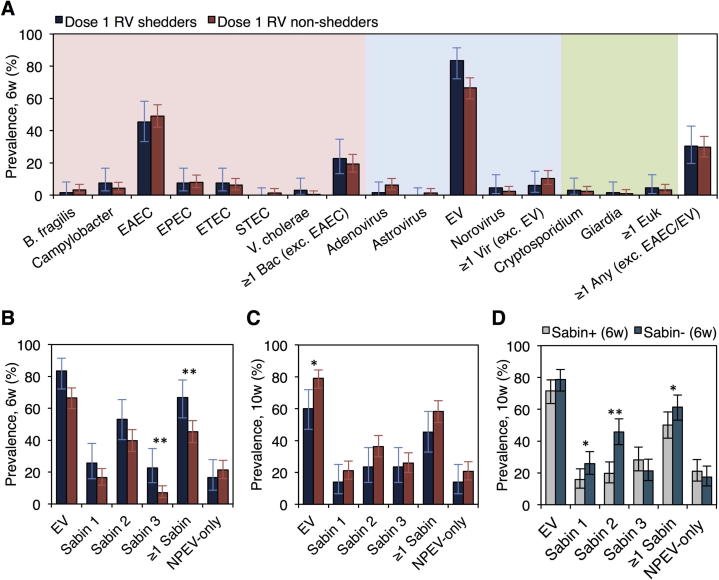
Association between concurrent pathogens and Rotarix replication. (A) Prevalence of concurrent pathogens at 6 weeks of age according to shedding status at 4 and/or 7 days after the first dose of RV1. Pathogens present in at least 1% of the study population are included. (B, C) Prevalence of Sabin viruses and NPEVs at 6 and 10 weeks of age according to shedding status. (D) Prevalence of enteroviruses at 10 weeks of age according to the shedding of ≥ 1 Sabin virus at 6 weeks. The shedding of Sabin viruses at 10 weeks of age was used as an indicator of take following the OPV dose administered at 6 weeks. ^*^P < .05; ^**^P < .005. Abbreviations: Bac, bacteria; EAEC, enteroaggregative *Escherichia coli*; EPEC, enteropathogenic *E. coli*; ETEC, enterotoxigenic *E. coli*; Euk, eukaryote; EV, enterovirus; NPEV, non-polio enterovirus; RV, rotavirus; Sabin+, positive for ≥1 Sabin serotype; Sabin-, negative for all Sabin serotypes; STEC, Shiga toxin-producing *E. coli*; Vir, virus; w, weeks.

Rotavirus shedding at >100 copies per reaction at 4 and/or 7 days following the first dose of RV1 was observed in 66/278 (24%) per-protocol infants with complete samples and no pre-vaccination shedding (Supplementary Fig. 2). Among shedders, 46/66 (70%) seroconverted, compared with 95/212 (45%) non-shedders (χ^2^, *P* < .001). Baseline characteristics were comparable between shedders and non-shedders (Supplementary Table 2), with the exception of rotavirus-specific serum IgA, which was detected in 8/66 (12%) shedders and 65/212 (31%) non-shedders (Fisher’s exact test, *P* = .002). We observed no association between intestinal bacteria, eukaryotes, mixed infections, concurrent diarrhea, or pathogen count at 6 weeks and the prevalence of shedding (*P* values >.05; Fig. 2A; Supplementary Table 3). Enterovirus abundance at 6 weeks was significantly greater in shedders than non-shedders (Ct, 28.8 ± 4.3 vs 30.5 ± 4.3; Wilcoxon’s rank sum, FDR-corrected *P* = .046; Supplementary Table 3) – a discrepancy attributable in part to a greater prevalence of Sabin viruses (Fig. 2B). In addition, shedding of rotavirus appears to be associated with the diminished replication of co-administered OPV, since enterovirus prevalence at 10 weeks of age (i.e., 4 weeks later) was lower in rotavirus shedders than non-shedders (39/65 [60%] vs 166/210 [79%]; χ^2^, *P* = .014; Fig. 2C) – a trend that was also evident among Sabin viruses (29/64 [45%] vs 122/209 [58%]; χ^2^, *P* = .066). Nonetheless, after two doses rotavirus seroconversion did not differ by type 3 poliovirus seroconversion status (146/447 [33%] seroconverted among poliovirus sero-responders and 26/103 [25%] among non-responders; χ^2^, *P* = .143). Poliovirus shedding at 10 weeks was significantly lower among individuals shedding poliovirus at 6 weeks (Fig. 2D).

### 3.4 Association between bacterial microbiota composition and RV1 outcome

**Fig. 3:**
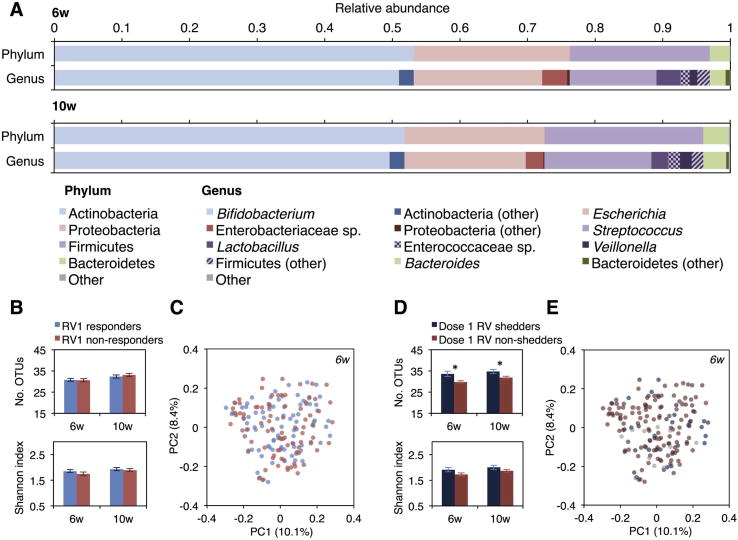
Association between microbiota composition and Rotarix response. (A) Phylum- and genus-level composition of the bacterial microbiota at 6 and 10 weeks of age. (B) OTU count and Shannon index (mean ± standard error) by rotavirus seroconversion status. (C) Unweighted Unifrac distances between 6-week samples, visualized via principal coordinates analysis. (D, E) Equivalent alpha and beta diversity plots are displayed with respect to shedding status after the 6-week RV1 dose. ^*^P < .05. Abbreviations: OTU, 97%-identity operational taxonomic unit; PC, principal coordinate; RV, rotavirus; RV1, Rotarix; w, weeks.

We obtained an average of 25,254 (SD 13,091) sequences per sample, encompassing 153 OTUs. The composition of the microbiota was similar at 6 and 10 weeks (Fig. 3A), with a small number of dominant OTUs (Supplementary Fig. 3).

Analyses of alpha and beta diversity are summarized in Supplementary Table 4. We observed no significant differences in microbiota diversity (Fig. 3B), stability (Supplementary Fig. 4), or taxon relative abundance (non-parametric *t* test, FDR-corrected *P* values >.15; Supplementary Table 5) according to seroconversion status, and no significant clustering of samples based on Unifrac distances (adonis, *P* values >.05; Fig. 3C; Supplementary Fig. 5). Infants who shed rotavirus 4 and/or 7 days after the 6-week RV1 dose harbored a greater number of OTUs before vaccination (linear regression, *P* = .007; Fig. 3D). We also observed a significant difference in pre-vaccination microbiota composition according to shedding status based on unweighted Unifrac (adonis, *P* = .032; Fig. 3E), but this accounted for a very small proportion of the variance among samples (R^2^ = 0.012). Rotavirus shedding was not associated with microbiota stability between 6 and 10 weeks (Supplementary Fig. 4C), nor did we observe any differences in pre-vaccination taxon abundance between shedders and non-shedders (non-parametric *t* test, FDR-corrected *P* values >.15). At 10 weeks, a modest enrichment of the phyla Bacteroidetes and Verrucomicrobia was observed among infants who shed rotavirus following the 6-week RV1 dose (Supplementary Table 6).

Random Forest models based on OTU abundance data failed to accurately predict rotavirus seroconversion (mean accuracy 43.4% and 45.7% at 6 and 10 weeks, respectively; baseline accuracy, 50.6%; *P* values >.05), but showed modest predictive accuracy for shedding after dose 1 (mean accuracy 60.3% and 60.8% based on OTUs measured at 6 and 10 weeks, respectively; baseline accuracy, 50.0%; *P* = .038 and .040; Supplementary Fig. 6).

### 3.5 Impact of probiotics on the bacterial microbiota

**Fig. 4:**
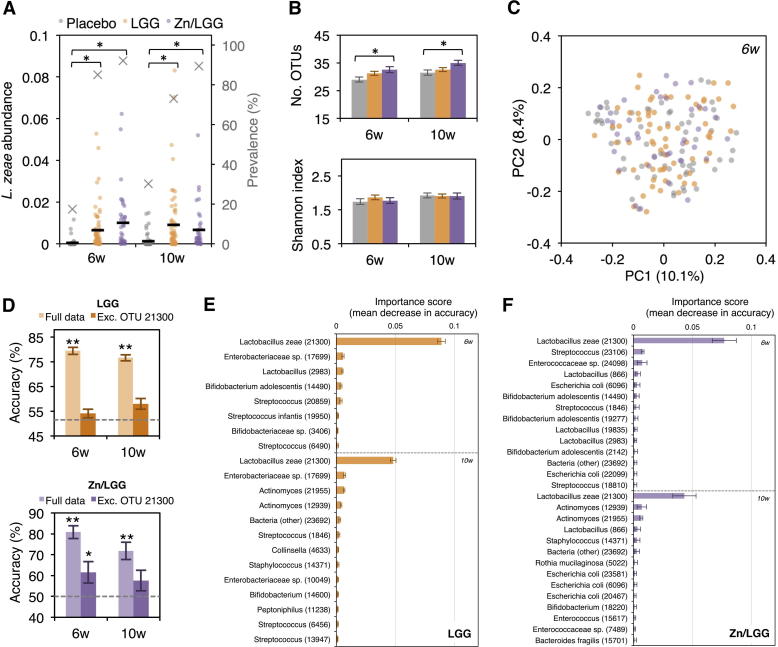
Impact of probiotic supplements on the bacterial microbiota. (A) Receipt of probiotics resulted in enrichment of a single OTU (classified as *L. zeae*). Mean relative abundance of this OTU in each study arm is indicated by a horizontal line, while prevalence is indicated by a cross. (B) OTU count and Shannon index (mean ± standard error) by study arm. (C) Unweighted Unifrac distances between 6-week samples, visualized via principal coordinates analysis. (D) Mean accuracy (±SD) across 100 iterations of the Random Forest algorithm for models predicting receipt of probiotics-only (upper) or zinc and probiotics (lower). OTU 21300 corresponds to the *Lactobacillus* strain that was enriched among probiotics recipients. (E, F) Highest-ranking taxa (and corresponding OTU IDs) by Random Forest importance score (mean decrease in accuracy ± SD) for models predicting receipt of (E) probiotics-only and (F) zinc and probiotics. ^*^P < .05; ^**^P < .005. Abbreviations: LGG, probiotics (*Lactobacillus rhamnosus* GG); OTU, 97%-identity operational taxonomic unit; PC, principal coordinate; w, weeks; Zn, zinc.

Our study was designed to compare microbiota composition according to seroconversion status, and we therefore included an equal number of responders and non-responders rather than a random sample from each study arm. However, since 16S microbiota composition was not strongly correlated with seroconversion status, we pursued an exploratory analysis of microbiota diversity and composition by study arm. Overall, the impact of study arm was modest (Fig. 4), as discussed further in the Supplementary Results. The probiotic strain appears to correspond to a single OTU, classified as *Lactobacillus zeae*, which was more prevalent and abundant in infants receiving the probiotic intervention (Fig. 4A). Among probiotic recipients, pre-vaccination abundance of this OTU was associated with rotavirus shedding after dose 1, but not seroconversion (Supplementary Fig. 7).

### 3.6 Sensitivity analyses

Sensitivity analyses are discussed in the Supplementary Results.

## 4 Discussion

Throughout early life, infants living in resource-poor settings are exposed to multiple, diverse enteropathogens. The negative repercussions of repeated pathogen exposure include deficits in growth [35], gut integrity [36], and OPV immunogenicity [8]. Among infants in south India, we observed a high prevalence of enteropathogens (albeit generally in the absence of symptoms). However, we did not observe an inhibitory effect of these enteropathogens on RV1. Indeed, infants harboring ≥1 bacterial pathogen during both doses were more likely to respond to this vaccine.

Rotavirus shedding following the 6-week vaccine dose was positively associated with rotavirus seroconversion but negatively correlated with the take of co-administered OPV. This observation is consistent with an inhibitory effect of OPV on RV1 (although we observed no inhibitory association between the immunogenicity of these vaccines after two doses) [37]. Given their potential to interfere with OPV [8], the presence of pathogenic bacteria at 6 weeks of age may perhaps have enhanced RV1 immunogenicity by inhibiting the replication of co-administered Sabin viruses. Alternatively, concurrent bacteria may have promoted RV1 immunogenicity via an adjuvant effect (e.g., through the induction of TLR signaling). It is worth noting, however, that the association between bacterial pathogens and RV1 response was contingent on the exclusion of EAEC, which we omitted from primary comparisons because of its high prevalence and limited association with diarrhea during previous studies in resource-poor settings using TACs [28]. Irrespective of whether EAEC was included, our findings do not support the view that bacterial pathogens impair the immunogenicity of RV1 – a conclusion consistent with recent findings from Bangladesh [9].

Infants who shed rotavirus after their 6-week RV1 dose exhibited a higher prevalence of enteroviruses at the time of vaccination. These enteroviruses can be attributed primarily to the residual replication of Sabin viruses administered at birth. Again, this observation may relate to the replication of Sabin viruses co-administered with RV1. The take of OPV given at 6 weeks was diminished among infants shedding Sabin viruses at that time, potentially reflecting an inhibitory effect of continued replication of the OPV birth dose or of vaccine-induced mucosal immunity. By either mechanism, existing Sabin polioviruses may have enhanced RV1 response by inhibiting the replication of co-administered OPV (conceptual model in Supplementary Fig. 8). Prior rotavirus exposure, inferred by the presence of rotavirus-specific serum IgA at baseline, has been linked with impaired RV1 immunogenicity in several previous studies [6], [38]. Here, IgA seropositivity at baseline was negatively correlated with dose 1 shedding but not with seroconversion after two doses.

We observed no differences in the composition or stability of the bacterial microbiota according to rotavirus seroconversion status. A greater OTU count and shift in overall community structure was apparent in individuals who shed rotavirus after the first RV1 dose. However, the size of this effect was modest and was not indicative of dysbiosis. These findings do not necessarily preclude a role of the intestinal microbiota in shaping broader geographic trends in RV1 immunogenicity. The composition of the microbiota among infants in this study is likely to differ considerably from that of infants in high-income countries [12]. Given the poor seroconversion rates observed in this trial (31%), it is possible that all infants harbored a bacterial community structure inhibitory to RV1 replication.

Our findings are at odds with a recent study of RV1 in Ghana, wherein infants who seroconverted exhibited a lower abundance of Bacteroidetes, a higher abundance of bacteria related to *Streptococcus bovis*, and a microbiota composition closer to that of Dutch infants compared with non-seroconverters [39]. The authors of that paper speculate that bacteria related to *S. bovis* may be more immunostimulatory than those in the Bacteroidetes phylum, potentially acting as an adjuvant to the rotavirus vaccine. However, we observed no significant differences in microbiota composition (including *Streptococcus* abundance) according to RV1 seroconversion and greater abundance of Bacteroidetes at 10 weeks of age among rotavirus shedders. These discrepancies may reflect differences in methodology (next-generation sequencing versus microarray) or baseline microbiota composition, and highlight the difficulties that are likely to be faced in identifying mechanistic links between the intestinal microbiota and oral vaccine outcome using observational data.

The administration of probiotics had a minimal impact on the intestinal microbiota of these infants, as illustrated by the failure of Random Forest models to accurately distinguish infants by study arm when the OTU corresponding to the probiotic strain was omitted. Despite the daily administration of 10^10^ organisms, the enriched OTU accounted for a mean relative abundance of <1% among probiotic recipients at 6 and 10 weeks, potentially reflecting passive transit rather than successful colonization.

Our study was limited by the lack of shedding data for the second RV1 dose. Although demography, growth, and several other baseline characteristics did not differ between the responders and non-responders included in this study, we did not consider several other potential confounders that may influence microbiota composition in early infancy, such as mode of delivery and antibiotic exposure [22], [40]. Factors such as primer selection and sample handling (e.g., freeze–thaw cycles) may have introduced bias into our assessment of microbiota composition [41], [42]. However, these were present across all samples and would therefore have influenced comparison groups equally. Finally, co-administration of OPV may have obscured a role for other enteric viruses, particularly NPEVs, in shaping RV1 immunogenicity. Further study among infants receiving inactivated poliovirus vaccine rather than OPV would allow the significance of NPEVs for RV1 immunogenicity to be tested.

Overall, our findings support a modest inhibitory effect of co-administered OPV on the first dose of RV1. However, we did not observe a greater pathogen burden among infants who failed to respond to RV1, nor did we observe any major differences in bacterial microbiota composition in these individuals. Future studies on a broader geographic and socioeconomic scale, or those considering different aspects of microbial community composition or function, may yet reveal an important role for the intestinal microbiota in shaping RV1 response.

## Funding

This work was funded by grants from the UK Medical Research Council, the Bill & Melinda Gates Foundation, the Government of India Department of Biotechnology (BT/PR-14943/FNS/20/484/2010), and PATH. The Imperial Biomedical Research Centre Genomics Facility, which is supported by funding from the National Institute for Health Research, provided resources and support that have contributed to the results reported within this paper.

## Conflict of interest

The authors declare no conflicts of interest.

## Data availability

16S rRNA sequences have been deposited in the European Nucleotide Archive (accession number PRJEB21946). An OTU table obtained after sequence assembly, quality filtering, chimera removal, taxonomic assignment, and minimum abundance filtering is provided alongside relevant metadata in the Supplementary Materials.
