# Supplementary material for: Influence of the intestinal microbiota on the immunogenicity of oral rotavirus vaccine given to infants in south India
Source: Vaccine. 2018 Jan 4;36(2):264–72. doi: 10.1016/j.vaccine.2017.11.031 (PMC5755003; doi:10.1016/j.vaccine.2017.11.031)
Supplement: Supplementary data 1 [file mmc1.docx]

Supplementary Methods

Study area and population

Chinnallapuram is a densely populated urban area in the southwest of Vellore. The most common occupation of residents is the production of ‘beedis’ – hand-made cigarettes consisting of tobacco wrapped in dried leaves [1]. Homes are predominantly built from permanent materials such as bricks and concrete, although ‘kutcha’ houses (made from mud brick, thatch, or other low-quality materials) are not uncommon [2]. Stunting was observed in approximately 40% of children at 2 years of age during a birth cohort study spanning Chinnallapuram and adjacent areas [1]. Microbial contamination of municipally supplied water is ubiquitous [3]. Close contact with animals, poor hand hygiene practices, overcrowding, and lack of access to functioning toilets may also contribute to the high incidence of enteric disease that is observed among infants [2, 4]. Caesarean section accounts for 6–18% of births [1, 5].

Trial design

Full details of the study design have been published elsewhere (CTRI/2012/05/002677; [ClinicalTrials.gov](http://ClinicalTrials.gov) identifier NCT01616693; Lazarus et al. [6]). Between 18 July 2012 and 22 February 2013, a total of 620 infants were recruited to the trial at 5 weeks of age. Participants were assigned at random to receive one of four treatment regimens: (i) supplements of zinc (5 mg of zinc sulphate heptahydrate) and probiotics (*Lactobacillus rhamnosus* GG capsules containing 10^10^ organisms) administered daily from 5 to 11 weeks of age; (ii) zinc supplements and probiotic placebo; (iii) probiotic supplements and zinc placebo; and (iv) zinc placebo and probiotic placebo. All participants received Rotarix (RV1) at 6 and 10 weeks of age, and trivalent OPV at 6, 10, and 14 weeks of age. Serum samples for the measurement of anti-poliovirus neutralizing antibodies and anti-rotavirus IgA antibodies were collected at 6 and 14 weeks of age, and stool samples were obtained on the day of and 4 and/or 7 days after each dose of RV1 and stored at -70°C. Response to OPV was defined as an increase in poliovirus serotype 3 neutralizing antibody titer from <1:8 to ≥1:8 or a four-fold rise in titer, assuming a decay in pre-vaccination titer with a half-life of 28 days. During the baseline (5-week) visit to the study clinic, information regarding the sociodemography and health status (e.g., vaccination history and breastfeeding status) of each infant was collected based on an interview with a parent or guardian. Anthropometric measurements were carried out at the 6- and 10-week vaccination visits.

Enteropathogen testing

**Nucleic acid extraction.** Extraction of DNA and RNA was performed from 200 mg of stool using the QIAamp DNA Stool Mini Kit (Qiagen). The Qiagen extraction protocol was modified to include an initial bead-beating step of 2–3 minutes using 500-μm glass beads, after which samples were heated to 90–95°C for 5 minutes. The final elution volume was 200 μl, of which 100 μl was transferred to a tube containing 100 μl of RNA storage solution (AM7001, Ambion). The resulting total nucleic acid samples were stored at -70°C until testing. Extrinsic controls for DNA (10^6^ copies of phocine herpesvirus [PhHV]) and RNA (10^7^ copies of MS2 bacteriophage) were spiked into the lysis buffers to monitor extraction and amplification efficiency. A negative extraction control was included in each batch to monitor cross-contamination.

Infants were excluded if either the 6- or 10-week stool sample lacked sufficient material for nucleic acid extraction. However, since the preparation of 200-mg aliquots, nucleic acid extraction, and TAC assays were carried out in tandem, there were several instances in which it became apparent that the 6- or 10-week stool sample collected from an infant lacked sufficient material for nucleic acid extraction only after the TAC assay for the corresponding pre-vaccination sample had been completed. In these instances, the completed assays were retained in the final analyses.

**TaqMan array card assay.** TaqMan array cards (TACs) consist of 384-well arrays of singleplex real-time reverse-transcription PCR (RT-PCR) assays that have demonstrated high sensitivity and specificity during enteropathogen detection [7]. Procedures and a complete list of pathogen targets for the TAC assay have previously been reported [8]. Diarrheagenic *Escherichia coli* subgroups were examined using multiple targets and defined as follows: enteroaggregative *E. coli* (EAEC), *aatA* and/or *aaiC*; enteropathogenic *E. coli* (EPEC), *eae* and/or *bfpA*; enterotoxigenic *E. coli* (ETEC), heat-stable enterotoxin and/or heat-labile enterotoxin; and Shiga toxin-producing *E. coli* (STEC), *stx1* and/or *stx2*. The presence of adenovirus was determined based on amplification of either the pan-adenovirus or serotype 40/41 targets, while norovirus was defined as the presence of either genogroup 1 or genogroup 2 targets. The presence of *Campylobacter, Giardia,* and *Cryptosporidium* was determined based on the amplification of genus- or species-specific targets. Based on these criteria, we distinguished between 31 distinct enteropathogens in each sample.

Samples were considered eligible for the analysis if they were positive for PhHV and at least one RNA target (MS2 or other). The extraction and assay were repeated for samples that failed to meet these criteria (requiring an additional freeze–thaw cycle for aliquoting). If repetition failed to yield an eligible assay, the sample was excluded from the analysis. The extraction batch was repeated if a pathogen target was present at a Ct of <35 in the negative extraction control. Throughout the procedure, the order in which samples were extracted and analyzed was randomized, and the nature of samples (responder vs non-responder, 6- vs 10-week sample, and study arm) was blinded. A no-template control was assessed approximately once per week to monitor laboratory contamination.

**Detection of Sabin polioviruses among enterovirus-positive samples.** We tested for the presence of Sabin polioviruses in all samples positive for the pan-enterovirus target in the TAC assay (with the exception of two enterovirus-positive 10-week samples). This was achieved using a one-step multiplex real-time RT-PCR assay for simultaneous detection of all three Sabin types that has previously been described [9]. Viral RNA was extracted from a 20% stool suspension in minimum essential medium using the QIAxtractor (Qiagen) – an automated nucleic acid purification system – with Vx reagents. Before extraction, each sample was spiked with an extrinsic internal control (1 μl of MS2 phage), which was used as a measure of extraction and amplification efficiency. The PCR primers and probes (Sabin 1, 2, and 3) were designed to amplify the variable VP1 region of the Sabin-type poliovirus genome. Ct cut-offs of 36 for serotype 1 and 37 for serotypes 2 and 3 were applied [9]. For samples that were negative for MS2, the extraction and PCR were repeated. Positive controls for each serotype, a negative control, and a no-template control were included in each run.

**Detection of rotavirus shedding.** Infants were included in the analysis of RV1 take if their 6-week pre-vaccination sample had been successfully assayed via TAC. RNA was extracted from 200 μl of 10% stool suspension (prepared in minimum essential medium) using the QIAxtractor with Vx reagents (Qiagen). Reverse transcription was performed with random primers (Pd[N]6 hexamers; Invitrogen) using Moloney murine leukemia virus reverse transcriptase enzyme (Superscript II MMLV RT, which lacks RNase H activity; Invitrogen). Real-time PCR was then used for quantitation of the VP6 gene of group A rotavirus. A plasmid containing the VP6 gene (1356 bp) of a rotavirus G1P[8] strain was cloned into a TOPO-TA 2.1 vector (3900 bp; Invitrogen), propagated in *E. coli* DH5α cells, and purified using the Plasmid Miniprep kit (Qiagen). For calibration of the assay, a standard curve was generated from a ten-fold dilution series representing between 10^8^ and 10^1^ copies of VP6 plasmid. Quantitative PCR was performed using the primers VP6-F (5’-GACGGVGCRAC-TACATGGT-3’) and VP6-R (5’-GTCCAATTCATNCCTGGTG-3’; Sigma–Aldrich), which amplify a 379-bp region of the VP6 gene [10], and the VP6 probe 5’-(FAM)-CCACCRAAY-ATGACRCCAGCNGTA-NFQ-MGB-3’ (Applied Biosystems) [11]. This assay was performed on an ABI PRISM 7500 thermal cycler (Applied Biosystems). The quantity of rotavirus-specific cDNA was calculated by normalizing the Ct values of stool samples with that of the internal VP6 plasmid standard controls. A standard curve was constructed by linear regression, which allowed the concentration of cDNA present in the samples to be converted to copy numbers per reaction using the ABI PRISM 7500 Software v2.0.6. Rotavirus shedding was defined as the presence of >100 viral copies per reaction either 4 or 7 days after vaccination. Infants shedding above this threshold on the day of vaccination were excluded.

V4-16S rRNA sequencing

Infants were considered for the microbiota subset if eligible TAC assays (positive for PhHV and at least one RNA target) had been performed for 6- and 10-week samples and if sufficient stool material for DNA extraction was available at both timepoints. During selection of the microbiota subset, we included all eligible individuals who received only placebos and all who received probiotics with zinc placebo. The remaining individuals were selected at random from recipients of both zinc and probiotics.

All laboratory and bioinformatic processing steps follow those described by Parker et al [12]**.** The order of samples for DNA extraction, PCR, and sequencing was randomized. Samples were split across two MiSeq runs, each containing 190 samples (180 samples, eight extraction controls, and two no-template controls in the first run; 160 samples, six extraction controls, two no-template controls, and 22 technical replicates in the second). Technical replicates included sequencing replicates (using the same PCR product; n = 8); PCR replicates (using different reverse primers on the same MiSeq run; n = 5); replicates of both PCR and sequencing (using different reverse primers on different MiSeq runs; n = 5); and amplicons obtained from template DNA extracted without enzyme incubation (included for exploratory purposes only; n = 5).

For the assessment of alpha and beta diversity, we generated ten separate rarefactions at 3,500 sequences per sample and calculated mean values for OTU count, Shannon index, and Unifrac distances. Proteobacteria-specific OTU counts were determined using the QIIME functions *filter_taxa_from_otu_table.py* and *alpha_diversity.py*.

Data analysis

**Baseline characteristics.** Baseline characteristics of infants were compared according to RV1 outcome (seroconversion/shedding) using Fisher’s exact test and Wilcoxon’s rank sum test for binary and continuous variables, respectively.

**Interim analysis and sample size calculation.** Our primary objective in this study was to examine the impact of harboring 1 or more enteropathogens at either 6 or 10 weeks of age on the odds of seroconverting to rotavirus. However, since the sensitive detection of multiple enteropathogens via TACs had not previously been conducted in this population, we used an interim, blinded analysis of enteropathogen prevalence to inform final sample size calculations. Herein, we selected 112 individuals (14 RV1 responders and 14 non-responders from each of the four study arms) and performed the extraction and TAC assay for the 6- and 10-week stool samples of this subset. Without unblinding study arm or RV1 outcome, the prevalence of enteropathogens at 6 and 10 weeks of age was determined. Five individuals were excluded at each timepoint owing to insufficient sample availability or the absence of RNA targets (including MS2) in the TAC assay. The interim analysis revealed a high prevalence of EAEC (67/107 [63%] at 6 weeks and 70/107 [65%] at 10 weeks) and enteroviruses (74/107 [69%] at 6 weeks and 78/107 [73%] at 10 weeks) – we subsequently confirmed that Sabin viruses accounted for the majority of the latter, as described in the main text. Rotavirus infections were observed in 3/107 (3%) 6-week samples and 1/107 (1%) 10-week samples. Infections other than EAEC, enterovirus, and rotavirus were observed in 48/107 (45%) and 44/107 (41%) of 6- and 10-week samples, respectively, and were present during at least one of the two doses in 68/105 (65%) individuals. By contrast, when enteroviruses and EAEC were included, ≥1 enteropathogen was observed at 6 or 10 weeks in 104/105 (99%) infants.

In light of the high prevalence of EAEC and (Sabin) enteroviruses, as well as the limited association between EAEC and diarrhea in previous studies using analogous methods of pathogen detection [13, 14], we decided to omit these TAC targets from the primary outcome analyses. Rotavirus was also excluded from analyses of pathogen groups because natural infection can result in seroconversion and detection at 10 weeks may simply reflect shedding of RV1. We estimated that the inclusion of at least 150 RV1 responders and 150 non-responders would provide 80% power to detect a reduction in the odds of rotavirus seroconversion (with an OR of ≤0.5 and an α of 0.05) associated with the presence of ≥1 enteropathogen (excluding EAEC, enterovirus, and rotavirus) at 6 or 10 weeks of age, assuming an overall infection rate of 65% in a study population comprising 50% responders and 50% non-responders [15].

**Comparisons of taxon abundance.** To identify potential phylum-, class-, and OTU-level differences in taxon abundance according to vaccine outcome or study arm, we used a non-parametric *t* test (implemented in the R package *permute*) [16]. For each taxon, a distribution of 9,999 *t* statistics was generated by randomly permuting the outcome assigned to each sample. The non-parametric *P* value was then determined by calculating the proportion of permuted *t* statistics greater than or equal to the observed *t* statistic. Permutations were stratified by MiSeq run to account for potential run-to-run variation. Taxa were assessed if they were present in at least 2% of samples being compared. *P* values for each taxonomic rank (phylum, class, genus, or OTU) were adjusted via Benjamini–Hochberg false discovery rate (FDR) correction [17]. We also used this approach to compare the relative abundance of the probiotic-associated OTU according to RV1 outcome (seroconversion/shedding) among probiotic recipients (either with or without zinc).

Impact of zinc and/or probiotic supplementation on the intestinal microbiota. We prioritized the placebo-only and probiotics study arms in our 16S rRNA gene sequencing study to enable the impact of probiotic supplementation on the microbiota to be assessed as a secondary objective. Overall, we included infants from three study arms: placebo (n = 63), probiotic (n = 69), and zinc/probiotic (n = 38). To examine the impact of zinc and/or probiotic supplementation on the intestinal microbiota, we compared alpha diversity (via linear regression), beta diversity (using the adonis function), and relative taxon abundance (using the non-parametric *t* test described above) according to study arm. The placebo-only group served as baseline for these comparisons.

**Random Forests.** We fit Random Forest models to classify infants according to: (i) rotavirus seroconversion; (ii) rotavirus take; (iii) receipt of probiotics (probiotics only vs placebo only); and (iv) receipt of both supplements (zinc/probiotics vs placebo only) based on their intestinal microbiota [18]. Separate analyses were carried out for 6- and 10-week samples. Since the *Lactobacillus rhamnosus* GG probiotic strain appeared to correspond to a single OTU, we fit separate Random Forest models either with or without this OTU during analyses of study arm in order to clarify the impact of probiotic supplementation on taxa other than the administered strain. Models were implemented in the R package *randomForest*, with 1,000 trees per forest and all other parameters at their default values. A table of OTU relative abundances (determined at a depth of 3,500 sequences per sample) served as input, and OTUs were included as predictors if they were present in at least 2% of the samples being compared. For each comparison, mean out-of-bag classification accuracy (where accuracy = 100 – out-of-bag error rate) and variable importance scores were determined over 100 iterations of the algorithm (analogous to the approach adopted by Subramanian et al. [19]). Significance of the model fit was assessed by generating 999 null models in which class labels were randomly assigned to the input variables and determining the proportion of null models with a classification accuracy greater than the mean accuracy of the fitted model.

Since the tree-building process in Random Forests is tailored towards isolating the majority class, the algorithm has a tendency to perform poorly if classes are imbalanced (e.g., if the number of non-shedders greatly outweighs the number of shedders) [20]. Under-sampling of the majority class has been shown to improve classification accuracy of minority class members when applying several machine-learning algorithms [21], including Random Forests [20]. Accordingly, if the majority class contained more than 60% of observations (as was the case in the analysis of vaccine take and zinc/probiotics), a random subset of samples from the majority class equal in size to the minority class was selected for each of the 100 iterations of the Random Forest algorithm. Specifically, for each of the 100 iterations of the Random Forest algorithm during analyses of dose 1 RV1 take, we included the 37 rotavirus shedders from the microbiota subset and a randomly selected subset of 37 non-shedders, while for analyses of zinc/probiotic supplementation, we included the 38 recipients of both supplements from the microbiota subset and a randomly selected subset of 38 placebo-only recipients.

**Run-to-run variation.** In the 13 samples sequenced in both MiSeq runs, we compared the number of OTUs, Shannon index, and phylum-, class-, genus-, and OTU-level abundances between replicates using Wilcoxon’s signed rank test for paired data. During comparisons of taxon abundance, *P* values for each set of comparisons (phylum-, class-, genus-, or OTU-level) were adjusted via Benjamini–Hochberg FDR correction. The clustering of sequencing replicates based on weighted and unweighted Unifrac distances was visualized using principal coordinates analysis, and the proportion of variance explained by MiSeq run and technical replicate assessed using the adonis function – a non-parametric permutation-based test that partitions sums of squares within a distance matrix between variables and residuals in a manner equivalent to multivariate analysis of variance [22].

Sensitivity analyses. For analyses of RV1 outcome with respect to enteropathogen burden and 16S bacterial microbiota, we performed sensitivity analyses in which: (i) lower Ct cut-offs (30, 25, and 20) were used to assess the presence or absence of TAC targets; (ii) samples lacking amplification of MS2 were excluded (to examine the impact of variation in the extraction efficiency of RNA targets); (iii) individuals positive for rotavirus-specific IgA at baseline were excluded (to account for the potential impact of prior rotavirus exposure on the outcomes of concurrent infection); and (iv) infants who experienced a robust response to vaccination (defined as both a four-fold rise in serum IgA titer and a post-vaccination titer of >90 U/ml) were compared with those who were seronegative post-vaccination (titer <20 U/ml) or experienced a fall in antibody titer between pre- and post-vaccination samples. A post-vaccination IgA titer of >90 U/ml has previously been suggested as an indicator of sustained protection against severe rotavirus-associated gastroenteritis [23]. To assess the potential impact of study arm on the primary outcome comparison, we fit separate logistic regression models in which we added interaction terms between enteropathogen infection status and (i) zinc supplementation (zinc vs no zinc); (ii) probiotic supplementation (probiotics vs no probiotics); and (iii) study arm.

Supplementary Results

Impact of probiotics on the bacterial microbiota

A single OTU, classified as *Lactobacillus zeae*, was significantly more abundant among infants receiving probiotic compared with placebo at both 6 and 10 weeks of age (Figure 4A; Supplementary Table 7). Given the clear discrepancies in the prevalence and abundance of this OTU according to treatment group, it is reasonable to conclude that it corresponds to the probiotic strain (the Greengenes database used for taxonomic assignment in this study does not contain a specific sequence for *L. rhamnosus*). Beyond the addition of this OTU, the influence of study arm on the bacterial microbiota was modest. Compared with placebo-only recipients, an increase in OTU count at 6 and 10 weeks of age was observed in recipients of zinc and probiotics, but not of probiotics alone (Figure 4B; Supplementary Table 4). No significant differences in Shannon index were observed according to study arm (Figure 4B). Similarly, receipt of both supplements, but not of probiotics alone, was associated with an increase in microbiota stability compared with placebo-only recipients, as inferred by a reduction in Unifrac distances between samples collected from the same infant over time in this study arm (Wilcoxon’s rank sum, *P* values 0.049 and 0.070 for comparisons of unweighted and weighted Unifrac distances, respectively, between zinc/probiotic recipients and placebo-only recipients; Supplementary Figure 4D). A shift in microbiota composition compared with the placebo-only group was observed among recipients of probiotics (with or without zinc) based on unweighted Unifrac distances (Figure 4C), but accounted for only a small proportion of variance among samples (R^2^ <0.03 for all comparisons). No significant clustering was apparent by study arm when using weighted Unifrac distances (adonis, *P* values >0.05 for all comparisons).

Aside from enrichment of the probiotic strain, few discrepancies in relative abundance distinguished placebo-only recipients from those who received supplements (Supplementary Table 7); however, two OTUs – one in the genus *Streptococcus* and the other in the family Enterococcaceae – were significantly more abundant at 6 weeks of age among infants who received both zinc and probiotics, while an OTU in the genus *Actinomyces* was significantly more abundant at 10 weeks of age among infants who received either probiotics alone or zinc and probiotics.

The outputs of Random Forests were consistent with these findings (Figure 4D). Models predicted the receipt of probiotics-only versus placebo-only with a mean accuracy of 79.4% and 76.7% at 6 and 10 weeks, respectively (baseline accuracy: 51.5%) – a significant improvement in fit compared with null models (*P* values <0.005). Predictive accuracy was similar when distinguishing recipients of zinc and probiotics from placebo-only recipients (80.0% and 71.8% at 6 and 10 weeks, respectively; baseline accuracy: 50.0%; *P* values <0.005). However, when separate models were fit in which the OTU corresponding to the probiotic strain was excluded, this predictive capacity was largely abrogated (Figure 4D). In line with these findings, the probiotic strain had a mean importance score that far outweighed that of any other OTUs (Figures 4E and 4F). Together, these findings support the notion that *L. rhamnosus* GG supplementation in these infants did not substantially alter the composition of the bacterial microbiota.

Sensitivity analyses

Threshold cycle. When lower Ct cut-off values were adopted for pathogen detection (corresponding to higher copy numbers of the RNA/DNA targets), the primary outcome was unchanged (Supplementary Table 8). We observed a significantly greater prevalence of enteroviruses in Rotarix responders than non-responders at 6 weeks of age (Ct 30, 89/156 [57%] vs 57/157 [36%]; χ^2^, *P* <0.001; Supplementary Figure 9) – a finding consistent with the analyses of pathogen abundance described above (Supplementary Table 1) – and a decrease in the prevalence of EAEC at this timepoint (Ct 20, 10/156 [6%] vs 23/157 [15%]; χ^2^, *P* = 0.018). Other associations were largely unchanged, although the increased proportion of responders infected with ≥1 pathogen at both doses observed using a Ct cut-off of 35 was no longer significant at lower thresholds.

Antibody titer. Among per-protocol infants included in the enteropathogen survey, 67 experienced a four-fold rise in rotavirus-specific serum IgA titer and had a post-vaccination titer in excess of 90 U/ml. When we compared these individuals to those who were seronegative post-vaccination or experienced a fall in antibody titer, associations between TAC targets and vaccine outcome were similar to those observed using standard seroconversion criteria (Supplementary Table 8) – these ‘robust responders’ were more likely than non-responders to harbor ≥1 bacterial enteropathogen (excluding EAEC) at 6 weeks of age (17/67 [25%] vs 17/136 [13%]; χ^2^, *P* = 0.021), and a borderline-significant increase in the likelihood of being infected with ≥1 enteropathogen at both doses (largely driven by bacterial infections) was observed in these individuals (13/67 [19%] vs 35/133 [10%]; χ^2^, *P* = 0.056). No significant associations were observed between viral or eukaryotic TAC targets and vaccine outcome as defined in this way. Similarly, comparisons of alpha and beta diversity did not reveal any significant discrepancies in the composition or diversity of the bacterial microbiota among robust responders versus non-responders (Supplementary Table 4).

Treatment group interactions. In the primary outcome analysis, we assessed the impact of harboring ≥1 enteropathogen at either 6 or 10 weeks on the odds of rotavirus seroconversion via logistic regression. We fit separate logistic regression models to examine potential interactions between enteropathogen infection status and treatment group (zinc supplementation, probiotic supplementation, or study arm). No significant interaction terms were observed (*P* values >0.05), suggesting that the association between concurrent infections and rotavirus seroconversion was not influenced by treatment group.

Other sensitivity analyses. The observed associations between concurrent enteropathogens and rotavirus seroconversion status were largely unchanged by excluding samples negative for MS2 (the extrinsic RNA control) or excluding individuals who were positive for rotavirus-specific serum IgA at baseline (observed in 167/551 [30%] infants who completed the trial per protocol) (Supplementary Table 8). In both instances, responders were significantly more likely than non-responders to harbor ≥1 enteropathogen (excluding EAEC and enterovirus) at both doses (χ^2^, *P* values <0.05) – an effect attributable primarily to discrepancies in the prevalence of bacterial infections, which were significantly more common among responders at 6 weeks of age (χ^2^, *P* values <0.05). When considering MS2-positive samples, non-responders were more likely than responders to harbor EAEC at both doses (50/128 [39%] vs 33/121 [27%]; χ^2^, *P* = 0.049) – a result that was also apparent in the primary analysis when using a Ct cut-off of 30 (Supplementary Figure 9). Likewise, comparisons of alpha and beta diversity were largely unaltered by the exclusion of infants positive for rotavirus-specific serum IgA at baseline (Supplementary Table 4).

Comparison of technical replicates

Thirteen samples were sequenced in both MiSeq runs. Measures of alpha diversity were generally consistent among these technical replicates (Supplementary Figures 10A and 10B), although a decrease in Shannon index was evident among samples in the second MiSeq run when assessed by Wilcoxon’s signed-rank test (*P* <0.001). No significant shift in beta diversity was evident according to MiSeq run (Supplementary Table 4). As would be expected, replicability was generally higher among samples in which the same PCR product was sequenced than those in which PCR was repeated (Supplementary Figures 10C and 10D). Technical replicate ID accounted for the majority of variance among these 26 samples based on Unifrac distances (adonis, R^2^, 0.858 and 0.987 for unweighted and weighted distances, respectively). Significant differences in relative abundance of the phyla Actinobacteria and Firmicutes were evident according to MiSeq run, although no individual OTU-level differences were apparent after FDR correction (Supplementary Table 9). While these observations point to the potential influence of run-to-run variation on our findings, the effect appears to be modest. Moreover, we accounted for potential batch effects during the design of this study by randomizing the order in which samples were assayed, and during statistical analyses via the inclusion of MiSeq run as a covariate in regression models and a constraint in permutation-based tests. It is therefore unlikely that the observed run-to-run variation substantially altered the outcomes of this study.

Negative controls

Combining across the 14 extraction controls and four no-template controls included in the two sequencing runs, we obtained a total of 58 sequences at the end of the analysis pipeline (3.2 sequences per control sample on average), suggesting that any contamination of laboratory reagents or cross-contamination during sample handling was negligible. Among these sequences, 53 (91%) corresponded to the five most abundant OTUs among fecal samples.

References

[1] Kattula D, Sarkar R, Sivarathinaswamy P, Velusamy V, Venugopal S, Naumova EN, et al. The first 1000 days of life: prenatal and postnatal risk factors for morbidity and growth in a birth cohort in southern India. BMJ Open 2014;4(7):e005404.

[2] Sarkar R, Sivarathinaswamy P, Thangaraj B, Sindhu KN, Ajjampur SS, Muliyil J, et al. Burden of childhood diseases and malnutrition in a semi-urban slum in southern India. BMC Public Health 2013;13:87.

[3] Brick T, Primrose B, Chandrasekhar R, Roy S, Muliyil J, Kang G. Water contamination in urban south India: household storage practices and their implications for water safety and enteric infections. Int J Hyg Environ Health 2004;207:473-80.

[4] Collinet-Adler S, Babji S, Francis M, Kattula D, Premkumar PS, Sarkar R, et al. Environmental factors associated with high fly densities and diarrhea in Vellore, India. Appl Environ Microbiol 2015;81:6053-8.

[5] Gladstone BP, Muliyil JP, Jaffar S, Wheeler JG, Le Fevre A, Iturriza-Gomara M, et al. Infant morbidity in an Indian slum birth cohort. Arch Dis Child 2008;93:479-84.

[6] Lazarus RP, John J, Shanmugasundaram E, Rajan AK, Thiagarajan S, Giri S, et al. A randomized, double blind, placebo controlled trial of supplementation with zinc and/or probiotics to enhance the immune response of oral rotavirus and polio vaccines in Indian infants*.* Vaccine 2017;epub ahead of print.

[7] Liu J, Gratz J, Amour C, Kibiki G, Becker S, Janaki L, et al. A laboratory-developed TaqMan Array Card for simultaneous detection of 19 enteropathogens. J Clin Microbiol 2013;51:472-80.

[8] Grassly NC, Praharaj I, Babji S, Kaliappan SP, Giri S, Venugopal S, et al. The effect of azithromycin on the immunogenicity of oral poliovirus vaccine: a double-blind randomised placebo-controlled trial in seronegative Indian infants. Lancet Infect Dis 2016;6:905-14.

[9] Taniuchi M, Begum S, Uddin MJ, Platts-Mills JA, Liu J, Kirkpatrick BD, et al. Kinetics of poliovirus shedding following oral vaccination as measured by quantitative reverse transcription-PCR versus culture. J Clin Microbiol 2015;53:206-11.

[10] Iturriza Gomara M, Wong C, Blome S, Desselberger U, Gray J. Molecular characterization of VP6 genes of human rotavirus isolates: correlation of genogroups with subgroups and evidence of independent segregation. J Virol 2002;76:6596-601.

[11] EuroRotaNet. European rotavirus detection and typing methods. Available from: http://www.eurorota.net/docs.php [last accessed: 24 November 2016].

[12] Parker EPK, Praharaj I, John J, Kaliappan SP, Kampmann B, Kang G, et al. Changes in the intestinal microbiota following the administration of azithromycin in a randomised placebo-controlled trial among infants in south India. Sci Rep 2017: in press.

[13] Taniuchi M, Sobuz SU, Begum S, Platts-Mills JA, Liu J, Yang Z, et al. Etiology of diarrhea in Bangladeshi infants in the first year of life analyzed using molecular methods. J Infect Dis 2013;208:1794-802.

[14] Platts-Mills JA, Gratz J, Mduma E, Svensen E, Amour C, Liu J, et al. Association between stool enteropathogen quantity and disease in Tanzanian children using TaqMan array cards: a nested case-control study. Am J Trop Med Hyg 2014;90:133-8.

[15] Dupont WD, Plummer WD Jr. Power and sample size calculations. A review and computer program. Control Clin Trials 1990;11:116-28.

[16] Simpson GL, Bates DM, Oksanen J. permute: functions for generating restricted permutations of data. R package version 0.9-4. 2016.

[17] Benjamini Y, Hochberg Y. Controlling the false discovery rate: a practical and powerful approach to multiple testing. J R Statist Soc B 1995;57:289-300.

[18] Breiman L. Random Forests. Mach Learn 2001;45:5-32.

[19] Subramanian S, Huq S, Yatsunenko T, Haque R, Mahfuz M, Alam MA, et al. Persistent gut microbiota immaturity in malnourished Bangladeshi children. Nature 2014;510:417-21.

[20] Chen C, Liaw A, Breiman L. Using Random Forest to learn imbalanced data. Available from: http://statistics.berkeley.edu/sites/default/files/tech-reports/666.pdf [last accessed: 26 May 2016].

[21] Drummond C, Holte RC. C4.5, class imbalance, and cost sensitivity: why under-sampling beats over-sampling. Workshop on Learning from Imbalanced Datasets II, ICML, Washington DC, 2003.

[22] Oksanen J, Blanchet FG, Friendly M, Kindt R, Legendre P, McGlinn D, et al. vegan: community ecology package. R package version 2.4-1. 2016.

[23] Patel M, Glass RI, Jiang B, Santosham M, Lopman B, Parashar U. A systematic review of anti-rotavirus serum IgA antibody titer as a potential correlate of rotavirus vaccine efficacy. J Infect Dis 2013;208:284-94.
