# Supplementary material for: Influence of the intestinal microbiota on the immunogenicity of oral rotavirus vaccine given to infants in south India
Source: Vaccine. 2018 Jan 4;36(2):264–72. doi: 10.1016/j.vaccine.2017.11.031 (PMC5755003; doi:10.1016/j.vaccine.2017.11.031)
Supplement: Supplementary data 2 [file mmc2.pdf]

Supplementary Table 1. Prevalence and abundance of enteropathogens by rotavirus seroconversion status.

	6 weeks								10 weeks							
	Prevalence, n (%)				Abundance, mean Ct ± s.d.				Prevalence, n (%)				Abundance, mean Ct ± s.d.			
Target	sero+ (n = 156)	sero- (n = 157)	*P*	FDR *P*	sero+ (n = 156)	sero- (n = 157)	*P*	FDR *P*	sero+ (n = 158)	sero- (n = 158)	*P*	FDR *P*	sero+ (n = 158)	sero- (n = 158)	*P*	FDR *P*
Aeromonas	1 (0.6)	0 (0.0)	0.498						1 (0.6)	3 (1.9)	0.623	0.962	35.0 ± 0.0	35.0 ± 0.2	0.312	0.587
Bacteroides fragilis	6 (3.8)	2 (1.3)	0.173	0.369	34.8 ± 1.3	35.0 ± 0.5	0.149	0.296	6 (3.8)	2 (1.3)	0.283	0.919	34.7 ± 1.5	34.9 ± 1.0	0.152	0.509
Campylobacter[a]	11 (7.1)	3 (1.9)	0.031	0.193	34.7 ± 1.7	34.9 ± 0.7	0.029	0.120	9 (5.7)	7 (4.4)	0.608	0.962	34.5 ± 2.3	34.6 ± 1.9	0.606	0.774
Clostridium difficile	0 (0.0)	3 (1.9)	0.248						0 (0.0)	2 (1.3)	0.498					
EAEC[a]	73 (46.8)	77 (49.0)	0.690	0.759	30.7 ± 6.0	30.0 ± 6.7	0.371	0.408	86 (54.4)	94 (59.5)	0.363	0.945	29.8 ± 6.2	28.5 ± 6.5	0.137	0.509
EPEC[a]	15 (9.6)	8 (5.1)	0.125	0.369	34.4 ± 2.3	34.6 ± 1.8	0.129	0.296	13 (8.2)	20 (12.7)	0.198	0.857	34.4 ± 2.4	34.1 ± 2.8	0.196	0.509
ETEC[a]	13 (8.3)	7 (4.5)	0.161	0.369	34.4 ± 2.2	34.7 ± 1.7	0.162	0.296	16 (10.1)	9 (5.7)	0.145	0.857	34.2 ± 2.8	34.5 ± 2.5	0.157	0.509
Salmonella	0 (0.0)	1 (0.6)	1.000						1 (0.6)	0 (0.0)	1.000					
Shigella/EIEC	0 (0.0)	0 (0.0)	1.000						1 (0.6)	0 (0.0)	1.000					
STEC[a]	0 (0.0)	3 (1.9)	0.248						1 (0.6)	2 (1.3)	1.000					
Vibrio cholerae	2 (1.3)	1 (0.6)	0.623						0 (0.0)	0 (0.0)	1.000					
Adenovirus[a]	6 (3.8)	11 (7.0)	0.217	0.369	34.9 ± 0.6	34.8 ± 1.2	0.231	0.363	10 (6.3)	13 (8.2)	0.516	0.962	34.6 ± 2.2	34.8 ± 0.7	0.539	0.774
Astrovirus	1 (0.6)	3 (1.9)	0.623	0.759	35.0 ± 0.3	34.7 ± 2.0	0.315	0.396	4 (2.5)	3 (1.9)	1.000	1.000	34.9 ± 1.1	34.8 ± 1.3	0.719	0.779
Enterovirus	113 (72.4)	104 (66.2)	0.235	0.369	29.5 ± 4.5	31.0 ± 4.1	0.004	0.042	119 (75.3)	118 (74.7)	0.897	1.000	29.5 ± 4.3	29.3 ± 4.4	0.879	0.879
Norovirus[a]	9 (5.8)	2 (1.3)	0.035	0.193	34.6 ± 2.1	34.8 ± 1.4	0.033	0.120	6 (3.8)	3 (1.9)	0.666	0.962	34.7 ± 1.7	34.8 ± 1.4	0.316	0.587
Rotavirus[a]	1 (0.6)	3 (1.9)	0.623	0.759	34.9 ± 0.6	35.0 ± 0.2	0.324	0.396	12 (7.6)	4 (2.5)	0.069	0.857	34.8 ± 1.1	35.0 ± 0.4	0.040	0.509
Sapovirus	1 (0.6)	0 (0.0)	0.498						2 (1.3)	3 (1.9)	1.000	1.000	35.0 ± 0.2	35.0 ± 0.3	0.655	0.774
Cryptosporidium[a]	3 (1.9)	4 (2.5)	1.000	1.000	34.9 ± 1.3	34.9 ± 1.3	0.715	0.715	3 (1.9)	2 (1.3)	1.000	1.000	35.0 ± 0.3	34.9 ± 1.4	0.655	0.774
Giardia[a]	0 (0.0)	3 (1.9)	0.248						1 (0.6)	1 (0.6)	1.000					

FDR correction and comparisons of abundance were carried out for pathogens present in at least 1% of the study population. Among samples positive for EPEC, 6/23 (26.1%) and 4/33 (12.1%) at 6 and 10 weeks, respectively, harbored both *eae* and *bfpA* (typical EPEC); the remainder were positive for *eae* alone (atypical EPEC). Abbreviations: Ct, threshold cycle; EAEC, enteroaggregative *Escherichia coli*; EIEC, enteroinvasive *E. coli*; EPEC, enteropathogenic *E. coli*; ETEC, enterotoxigenic *E. coli*; FDR, adjusted by Benjamini–Hochberg false discovery rate correction; s.d., standard deviation; sero+, responders; sero-, non-responder; STEC, Shiga toxin-producing *E. coli.*

[a] For enteropathogens assessed using multiple targets and/or in duplicate, the minimum Ct value was used for comparisons of pathogen abundance.

Supplementary Table 2. Baseline characteristics (shedders vs non-shedders).

	Enteropathogen subset			Microbiota subset		
	Shedders (n = 66)	Non-shedders (n = 212)	*P*	Shedders (n = 37)	Non-shedders (n = 116)	*P*
Treatment group						
Placebo	16 (24.2)	49 (23.1)	0.522	14 (37.8)	44 (37.9)	0.083
Zinc	13 (19.7)	46 (21.7)		0 (0.0)	0 (0.0)	
Probiotics	12 (18.2)	54 (25.5)		10 (27.0)	50 (43.1)	
Zinc/probiotics	25 (37.9)	63 (29.7)		13 (35.1)	22 (19.0)	
Age at enrollment (days)	35.8 (1.6)	35.8 (2.0)	0.344	35.9 (1.5)	36.0 (2.1)	0.455
Female	38 (57.6)	111 (52.4)	0.483	20 (54.1)	61 (52.6)	1.000
Mother's education						
None	5 (7.6)	12 (5.7)	0.322	4 (10.8)	4 (3.4)	0.032
Primary	15 (22.7)	32 (15.1)		10 (27.0)	12 (10.3)	
Secondary	34 (51.5)	116 (54.7)		17 (45.9)	65 (56.0)	
Higher secondary	9 (13.6)	28 (13.2)		4 (10.8)	20 (17.2)	
Degree/diploma	3 (4.5)	24 (11.3)		2 (5.4)	15 (12.9)	
House type						
Kutcha (temporary materials)	6 (9.1)	11 (5.2)	0.324	4 (10.8)	6 (5.2)	0.425
Mixed	28 (42.4)	81 (38.2)		14 (37.8)	43 (37.1)	
Pucca (permanent materials)	32 (48.5)	120 (56.6)		19 (51.4)	67 (57.8)	
Health status						
Any breastfeeding at enrollment	66 (100)	212 (100)	1.000	37 (100)	116 (100)	1.000
Positive for rotavirus IgA at baseline	8 (12.1)	65 (30.7)	0.002	3 (8.1)	36 (31.0)	0.005
Diarrhea at 6 weeks	3 (4.5)	8 (3.8)	0.726	2 (5.4)	3 (2.6)	0.595
Stunted at 6 weeks	13 (19.7)	38 (17.9)	0.719	9 (24.3)	15 (12.9)	0.120
Underweight at 6 weeks	7 (10.6)	29 (13.7)	0.675	5 (13.5)	13 (11.2)	0.770

Individuals are included in the shedding analyses if they completed the study per protocol, lacked rotavirus shedding pre-vaccination, and had eligible Taqman array card assays for the 6-week sample. Data are mean (standard deviation) or n (%). Shedders and non-shedders were compared using Wilcoxon's rank sum test or Fisher's exact test. Stunting was defined as a height-for-age *Z* score of < -2 and underweight as a weight-for-age *Z* score of < -2.

Supplementary Table 3. Prevalence and abundance of enteropathogens by dose 1 Rotarix take.

	6 weeks								10 weeks							
	Prevalence, n (%)				Abundance, mean Ct ± s.d.				Prevalence, n (%)				Abundance, mean Ct ± s.d.			
Target	shed+ (n = 66)	shed- (n = 212)	*P*	FDR *P*	shed+ (n = 66)	shed- (n = 212)	*P*	FDR *P*	shed+ (n = 65)	shed- (n = 210)	*P*	FDR *P*	shed+ (n = 65)	shed- (n=210)	*P*	FDR *P*
Aeromonas	0 (0.0)	1 (0.5)	1.000						1 (1.5)	3 (1.4)	1.000	1.000	35.0 ± 0.0	35.0 ± 0.1	0.962	0.962
Bacteroides fragilis	1 (1.5)	7 (3.3)	0.685	0.890	34.9 ± 0.7	34.8 ± 1.2	0.453	0.736	2 (3.1)	6 (2.9)	1.000	1.000	34.9 ± 0.8	34.8 ± 1.5	0.944	0.962
Campylobacter[a]	5 (7.6)	9 (4.2)	0.280	0.890	34.5 ± 2.0	34.8 ± 1.2	0.265	0.706	6 (9.2)	9 (4.3)	0.125	0.583	34.2 ± 2.9	34.7 ± 1.8	0.119	0.554
Clostridium difficile	0 (0.0)	1 (0.5)	1.000						0 (0.0)	0 (0.0)	1.000					
EAEC[a]	30 (45.5)	104 (49.1)	0.609	0.890	30.3 ± 6.5	30.6 ± 6.3	0.813	0.880	36 (55.4)	119 (56.7)	0.887	1.000	28.3 ± 7.1	29.4 ± 6.2	0.360	0.720
EPEC[a]	5 (7.6)	17 (8.0)	0.907	1.000	34.4 ± 2.5	34.5 ± 2.1	0.925	0.925	6 (9.2)	23 (11.0)	0.693	1.000	34.3 ± 2.4	34.2 ± 2.5	0.688	0.962
ETEC[a]	5 (7.6)	13 (6.1)	0.677	0.890	34.5 ± 2.2	34.6 ± 1.9	0.672	0.880	7 (10.8)	17 (8.1)	0.504	1.000	34.1 ± 3.0	34.3 ± 2.7	0.508	0.889
Salmonella	0 (0.0)	1 (0.5)	1.000						1 (1.5)	0 (0.0)	0.236					
Shigella/EIEC	0 (0.0)	0 (0.0)	1.000						0 (0.0)	1 (0.5)	1.000					
STEC[a]	0 (0.0)	3 (1.4)	1.000	1.000	35.0 ± 0.0	34.9 ± 1.0	0.335	0.706	1 (1.5)	2 (1.0)	0.556	1.000	34.9 ± 0.5	34.9 ± 0.6	0.695	0.962
Vibrio cholerae	2 (3.0)	1 (0.5)	0.141	0.865	35.0 ± 0.1	35.0 ± 0.4	0.082	0.533	0 (0.0)	0 (0.0)	1.000					
Adenovirus[a]	1 (1.5)	13 (6.1)	0.200	0.865	35.0 ± 0.0	34.8 ± 1.1	0.128	0.555	7 (10.8)	14 (6.7)	0.276	0.968	34.7 ± 1.6	34.7 ± 1.8	0.313	0.720
Astrovirus	0 (0.0)	3 (1.4)	1.000	1.000	35.0 ± 0.0	34.8 ± 1.6	0.335	0.706	2 (3.1)	5 (2.4)	0.670	1.000	34.8 ± 1.5	34.8 ± 1.2	0.759	0.962
Enterovirus	55 (83.3)	141 (66.5)	0.009	0.115	28.8 ± 4.3	30.5 ± 4.3	0.004	0.046	39 (60.0)	166 (79.0)	0.002	0.014	30.3 ± 4.8	29.3 ± 4.2	0.100	0.554
Norovirus[a]	3 (4.5)	5 (2.4)	0.400	0.890	34.9 ± 0.3	34.8 ± 1.7	0.380	0.706	1 (1.5)	6 (2.9)	1.000	1.000	34.8 ± 1.2	34.8 ± 1.5	0.563	0.720
Rotavirus[a]	1 (1.5)	1 (0.5)	0.419						10 (15.4)	3 (1.4)	<0.001	0.001	34.7 ± 1.1	34.9 ± 0.8	<0.001	<0.001
Sapovirus	0 (0.0)	1 (0.5)	1.000						1 (1.5)	3 (1.4)	1.000	1.000	35.0 ± 0.0	35.0 ± 0.3	0.962	0.962
Cryptosporidium[a]	2 (3.0)	5 (2.4)	0.672	0.890	34.8 ± 1.7	34.9 ± 1.2	0.764	0.880	0 (0.0)	5 (2.4)	0.595	1.000	35.0 ± 0.0	34.9 ± 1.2	0.212	0.720
Giardia[a]	1 (1.5)	2 (0.9)	0.558	0.890	35.0 ± 0.2	34.9 ± 0.7	0.706	0.880	0 (0.0)	1 (0.5)	1.000					

FDR correction and comparisons of abundance were carried out for pathogens present in at least 1% of the study population. Abbreviations: Ct, threshold cycle; EAEC, enteroaggregative *Escherichia coli*; EIEC, enteroinvasive *E. coli*; EPEC, enteropathogenic *E. coli*; ETEC, enterotoxigenic *E. coli*; FDR, adjusted by Benjamini–Hochberg false discovery rate correction; s.d., standard deviation; shed+, shedders; shed-, non-shedders; STEC, Shiga toxin-producing *E. coli.*

[a] For enteropathogens assessed using multiple targets and/or in duplicate, the minimum Ct value was used for comparisons of pathogen abundance.

Supplementary Table 4. Summary of microbiota diversity analyses.

Comparison	Age (w)	N[a]	N[b]	Number of OTUs			Number of OTUs (Proteobacteria)			Shannon index			Unweighted Unifrac		Weighted Unifrac	
				Effect size	SE	*P*	Effect size	SE	*P*	Effect size	SE	*P*	R^2	*P*	R^2	*P*
MiSeq run	-	13	13	-	-	0.152	-	-	-	-	-	<0.001	0.017	0.992	0.006	0.946
Study ID													0.635	<0.001	0.644	<0.001
RV1 seroconversion	6	85	83	0.078	1.066	0.942	0.026	0.313	0.935	0.072	0.092	0.436	0.006	0.605	0.003	0.733
	10	85	83	-0.612	1.029	0.553	-0.054	0.312	0.863	0.020	0.082	0.804	0.005	0.815	0.004	0.533
Dose 1 RV1 take	6	37	116	3.558	1.298	0.007	0.841	0.387	0.031	0.111	0.115	0.338	0.012	0.032	0.015	0.149
	10	37	116	2.759	1.234	0.027	0.896	0.387	0.022	0.164	0.101	0.105	0.015	0.003	0.006	0.452
Probiotics[c]	6	67	63	2.206	1.173	0.062	-	-	-	0.110	0.107	0.307	0.012	0.021	0.008	0.322
	10	67	63	1.105	1.178	0.350	-	-	-	-0.021	0.094	0.826	0.013	0.014	0.003	0.741
Zinc/probiotics[d]	6	38	63	3.576	1.483	0.018				0.008	0.127	0.952	0.016	0.027	0.025	0.072
	10	38	63	3.400	1.454	0.021				0.000	0.116	1.000	0.015	0.027	0.023	0.088
**Sensitivity analyses**																
RV1 seroconversion (titre >90 U/ml)	6	36	69	0.454	1.404	0.747	-	-	-	0.098	0.128	0.446	0.007	0.920	0.003	0.814
	10	36	69	0.159	1.380	0.909	-	-	-	0.016	0.111	0.887	0.007	0.868	0.008	0.423
RV1 seroconversion (IgA-)	6	67	57	0.017	1.228	0.989	-	-	-	-0.004	0.104	0.968	0.007	0.758	0.009	0.348
	10	67	57	-0.772	1.198	0.520	-	-	-	0.008	0.095	0.937	0.006	0.863	0.003	0.718

Abbreviations: IgA-, analysis restricted to individuals negative for rotavirus-specific IgA at baseline; OTU, 97%-identity operational taxonomic unit; RV1, Rotarix; SE, standard error; w, weeks.

[a] Number of responders/shedders/probiotics recipients (with/without zinc)/Miseq run 1 samples.

[b] Number of non-responders/non-shedders/placebo recipients/Miseq run 2 samples.

[c] Compares probiotics-only with placebo-only recipients.

[d] Compares zinc/probiotic recipients with placebo-only recipients.

Supplementary Table 5. Summary of taxon abundance comparisons according to Rotarix immunogenicity.

Taxonomic classification	OTU ID	Prevalence (%) sero+ (n = 85)	Prevalence (%) sero- (n = 83)	Relative abundance (mean % ± s.d.) sero+ (n = 83)	Relative abundance (mean % ± s.d.) sero- (n = 85)	*P*	FDR *P*
**6 weeks of age**							
**Phylum (all)**							
Bacteroidetes	-	58.8	47.0	3.471 ± 8.574	2.467 ± 7.599	0.4280	0.7792
Verrucomicrobia	-	2.4	2.4	0.029 ± 0.218	0.001 ± 0.010	0.5077	0.7792
Other	-	67.1	67.5	0.046 ± 0.053	0.041 ± 0.043	0.5283	0.7792
Firmicutes	-	100.0	100.0	21.345 ± 16.476	20.074 ± 15.475	0.6180	0.7792
Actinobacteria	-	100.0	100.0	52.480 ± 25.215	53.844 ± 27.578	0.7518	0.7792
Proteobacteria	-	100.0	100.0	22.629 ± 20.464	23.573 ± 24.424	0.7792	0.7792
**Class (FDR *P* <0.15)**							
NA							
**Genus (FDR *P* <0.15)**							
NA							
**OTU (FDR *P* <0.15)**							
NA							
**10 weeks of age**							
**Phylum (all)**							
Firmicutes	-	100.0	100.0	25.455 ± 20.175	21.417 ± 16.666	0.1556	0.9066
Verrucomicrobia	-	5.9	2.4	0.249 ± 2.023	0.022 ± 0.170	0.4253	0.9066
Actinobacteria	-	100.0	100.0	50.450 ± 24.419	53.200 ± 23.853	0.4533	0.9066
Proteobacteria	-	100.0	98.8	20.021 ± 19.299	21.445 ± 20.338	0.6369	0.9161
Other	-	65.9	67.5	0.047 ± 0.049	0.048 ± 0.051	0.8634	0.9161
Bacteroidetes	-	51.8	53.0	3.697 ± 9.437	3.867 ± 10.785	0.9161	0.9161
**Class (FDR *P* <0.15)**							
NA							
**Genus (FDR *P* <0.15)**							
NA							
**OTU (FDR *P* <0.15)**							
NA							

Abbreviations: FDR, adjusted by Benjamini–Hochberg false discovery rate correction; NA, not applicable; OTU, 97%-identity operational taxonomic unit; sero+, responders; sero-, non-responders.

Supplementary Table 6. Summary of taxon abundance comparisons according to dose 1 Rotarix take.

Taxonomic classification	OTU ID	Prevalence (%) shed+ (n = 37)	Prevalence (%) shed- (n = 116)	Relative abundance (mean % ± s.d.) shed+ (n = 37)	Relative abundance (mean % ± s.d.) shed- (n = 116)	*P*	FDR *P*
**6 weeks of age**							
**Phylum (all)**							
Verrucomicrobia	-	5.4	1.7	0.067 ± 0.329	0.001 ± 0.008	0.1027	0.6162
Other	-	78.4	64.7	0.053 ± 0.047	0.042 ± 0.049	0.2962	0.7923
Bacteroidetes	-	62.2	49.1	3.179 ± 6.532	2.107 ± 6.723	0.4023	0.7923
Firmicutes	-	100.0	100.0	18.978 ± 13.112	21.011 ± 17.015	0.5282	0.7923
Proteobacteria	-	100.0	100.0	23.640 ± 16.808	22.295 ± 24.128	0.7697	0.9236
Actinobacteria	-	100.0	100.0	54.083 ± 22.966	54.544 ± 27.457	0.9336	0.9336
**Class (FDR *P* <0.15)**							
NA							
**Genus (FDR *P* <0.15)**							
NA							
**OTU (FDR *P* <0.15)**							
NA							
**10 weeks of age**							
**Phylum (all)**							
Verrucomicrobia	-	13.5	1.7	0.600 ± 3.064	0.007 ± 0.053	0.0032	0.0192
Bacteroidetes	-	64.9	48.3	7.434 ± 14.917	2.730 ± 8.129	0.0089	0.0267
Proteobacteria	-	97.3	100.0	26.951 ± 22.371	19.062 ± 19.558	0.0464	0.0771
Firmicutes	-	100.0	100.0	17.923 ± 14.598	24.615 ± 19.488	0.0514	0.0771
Actinobacteria	-	100.0	100.0	47.052 ± 23.413	53.482 ± 24.765	0.1643	0.1972
Other	-	64.9	66.4	0.041 ± 0.040	0.046 ± 0.049	0.5724	0.5724
**Class (FDR *P* <0.15)**							
Verrucomicrobiae		13.5	1.7	0.600 ± 3.064	0.007 ± 0.053	0.0017	0.0187
Bacteroidia		64.9	48.3	7.434 ± 14.917	2.730 ± 8.129	0.0084	0.0462
Bacilli		100.0	100.0	15.212 ± 13.007	21.502 ± 17.998	0.0428	0.1287
Gammaproteobacteria		97.3	100.0	26.737 ± 22.476	18.990 ± 19.524	0.0468	0.1287
**Genus (FDR *P* <0.15)**							
Akkermansia		13.5	1.7	0.600 ± 3.064	0.007 ± 0.053	0.0025	0.1127
**OTU (FDR *P* <0.15)**							
Parabacteroides distasonis	23512	27.0	5.2	0.431 ± 1.242	0.011 ± 0.074	0.0003	0.0390
Bacteroides ovatus	9246	24.3	6.0	0.209 ± 0.607	0.017 ± 0.128	0.0020	0.1083
Akkermansia muciniphila	6041	13.5	1.7	0.600 ± 3.064	0.007 ± 0.053	0.0025	0.1083

Abbreviations: FDR, adjusted by Benjamini–Hochberg false discovery rate correction; NA, not applicable; OTU, 97%-identity operational taxonomic unit; shed+, shedders; shed-, non-shedders.

Supplementary Table 7. Summary of significant differences in taxon abundance according to study arm.

		Prevalence (%)			Relative abundance (mean % ± s.d.)			Placebo vs LGG		Placebo vs Zinc/LGG	
Taxonomic classification	OTU ID	Placebo (n = 63)	LGG (n = 67)	Zinc/LGG (n = 38)	Placebo (n = 63)	LGG (n = 67)	Zinc/LGG (n = 38)	*P*	FDR *P*	*P*	FDR *P*
**6 weeks of age**											
**Phylum/class (FDR *P* <0.15)**											
NA											
**Genus (FDR *P* <0.15)**											
Enterococcaceae sp.	-	58.7	-	76.3	0.577 ± 1.194	-	1.999 ± 4.170	-	-	0.0008	0.0376
**OTU (FDR *P* <0.15)**											
Lactobacillus zeae	21300	17.5	85.1	92.1	0.045 ± 0.182	0.659 ± 1.008	1.011 ± 1.407	0.0001	0.0136	0.0001	0.0126
Streptococcus	23106	12.7	-	34.2	0.004 ± 0.010	-	0.020 ± 0.032	-	-	0.0002	0.0126
Enterococcaceae sp.	24098	58.7	-	76.3	0.563 ± 1.174	-	1.981 ± 4.134	-	-	0.0011	0.0462
**10 weeks of age**											
**Phylum/class (FDR *P* <0.15)**											
NA											
**Genus (FDR *P* <0.15)**											
Enterobacteriaceae sp.	-	88.9	80.6	-	4.332 ± 8.289	1.316 ± 3.642	-	0.0039	0.0783	-	-
Peptoniphilus	-	15.9	4.5	-	0.018 ± 0.052	0.001 ± 0.006	-	0.0043	0.0783	-	-
Actinomyces	-	22.2	47.8	57.9	0.050 ± 0.131	0.158 ± 0.286	-	0.0047	0.0783	-	-
**OTU (FDR *P* <0.15)**											
Lactobacillus zeae	21300	30.2	73.1	89.5	0.116 ± 0.315	0.912 ± 1.540	0.674 ± 1.054	0.0001	0.0132	0.0001	0.0129
Actinomyces	21955	1.6	20.9	26.3	0.000 ± 0.004	0.013 ± 0.032	0.021 ± 0.039	0.0002	0.0132	0.0002	0.0129
Enterobacteriaceae sp.	17699	85.7	71.6	-	4.293 ± 8.244	1.302 ± 3.633	-	0.0050	0.1364	-	-
Streptococcus	1846	50.8	70.1	-	0.028 ± 0.034	0.049 ± 0.048	-	0.0054	0.1364	-	-
Peptoniphilus	11238	15.9	4.5	-	0.018 ± 0.052	0.001 ± 0.006	-	0.0043	0.1364	-	-
Enterobacteriaceae sp.	10049	28.6	13.4	-	0.022 ± 0.057	0.005 ± 0.013	-	0.0062	0.1364	-	-

Abbreviations: FDR, adjusted by Benjamini–Hochberg false discovery rate correction; LGG, probiotic (*Lactobacillus rhamnosus* GG); NA, not applicable; OTU, 97%-identity operational taxonomic unit.

Supplementary Table 8. Summary of sensitivity analyses.

Sensitivity analysis	Proportion with ≥1 pathogen at 6 or 10 weeks, n/N (%)		OR (95% CI)
	RV1 responders	RV1 non-responders	
Primary outcome	78/155 (67.8)	70/154 (45.5)	1.22 (0.78–1.90)
Ct 30	48/155 (31.0)	47/154 (30.5)	1.02 (0.63–1.66)
Ct 25	27/155 (17.4)	26/154 (16.9)	1.04 (0.57–1.88)
Ct 20	7/155 (4.5)	4/154 (2.6)	1.77 (0.51–6.19)
IgA titre >90 U/ml	33/67 (49.3)	58/133 (43.6)	1.26 (0.70–2.26)
MS2+	54/128 (42.1)	59/121 (48.8)	1.30 (0.79–2.15)
IgA-	51/110 (46.4)	57/114 (50.0)	1.16 (0.68–1.95)

Abbreviations: CI, confidence interval; Ct, threshold cycle; IgA-, analysis restricted to infants negative for rotavirus-specific IgA at baseline; MS2+, analysis restricted to infants with MS2-positive samples at 6 and 10 weeks of age; OR, odds ratio; RV1, Rotarix.

Supplementary Table 9. Differences in taxon abundance among technical replicates.

Taxonomic classification	OTU ID	Prevalence (%) Run 1 (n = 13)	Prevalence (%) Run 2 (n = 13)	Relative abundance (mean % ± s.d.) Run 1 (n = 13)	Relative abundance (mean % ± s.d.) Run 2 (n = 13)	*P*	FDR *P*
**Phylum (all)**							
Actinobacteria	-	100.0	100.0	49.877 ± 26.893	54.543 ± 27.722	0.0002	0.0015
Firmicutes	-	100.0	100.0	18.081 ± 11.563	15.114 ± 11.027	0.0007	0.0022
Bacteroidetes	-	38.5	53.8	6.866 ± 13.828	4.912 ± 10.448	0.0754	0.1508
Other	-	69.2	69.2	0.044 ± 0.045	0.026 ± 0.022	0.1169	0.1754
Proteobacteria	-	100.0	100.0	25.097 ± 21.435	25.371 ± 22.03	0.7354	0.8824
Verrucomicrobia	-	7.7	7.7	0.035 ± 0.127	0.033 ± 0.119	1.0000	1.0000
**Class (FDR *P* <0.15)**							
Bacilli	-	100.0	100.0	14.244 ± 12.690	11.490 ± 11.783	0.0002	0.0015
Actinobacteria	-	100.0	100.0	46.022 ± 28.969	49.600 ± 30.454	0.0002	0.0015
**Genus (FDR *P* <0.15)**							
Bifidobacterium	-	100.0	100.0	45.393 ± 28.751	49.004 ± 30.304	0.0011	0.0421
Streptococcus	-	100.0	100.0	9.908 ± 10.856	8.226 ± 10.360	0.0016	0.0421
**OTU (FDR *P* <0.15)**							
NA							

Phylum-, class-, genus-, and OTU-level differences in abundance among the 13 samples included in both MiSeq runs were assessed using Wilcoxon's signed rank test. Abbreviations: FDR, adjusted by Benjamini–Hochberg false discovery rate correction; NA, not applicable; OTU, 97%-identity operational taxonomic unit.

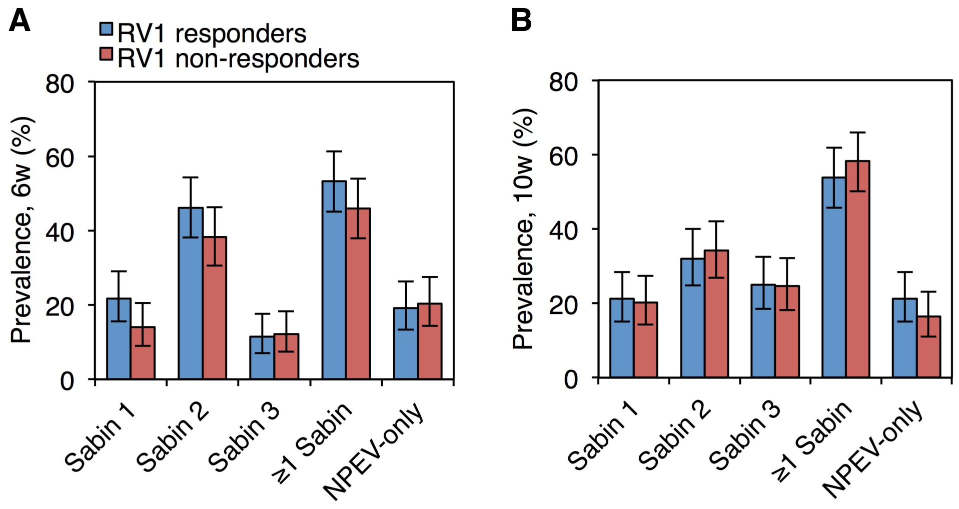

Supplementary Figure 1. Association between enterovirus subgroups and Rotarix immunogenicity. Data are displayed for Rotarix doses administered at (A) 6 weeks and (B) 10 weeks of age. Abbreviations: NPEV; non-polio enterovirus; RV1, Rotarix; w, weeks.

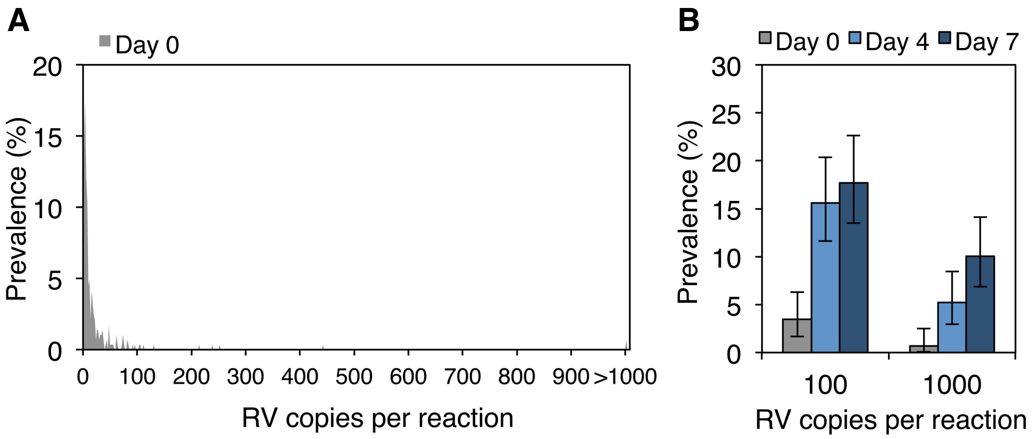

Supplementary Figure 2. Rotavirus shedding at 6 weeks of age. (A) Rotavirus shedding in pre-vaccination samples. Among the 313 per-protocol infants in which 6-week pre-vaccination samples were successfully assayed via TAC, the measurement of rotavirus shedding on the day of and 4 and 7 days after vaccination was carried out in 288 (92%) – stool samples were lacking at one or more timepoint for the remaining individuals. Rotavirus shedding at >100 copies per reaction on day 0 was observed in 10/288 (3%) individuals, who were excluded the final analyses of RV1 take. (B) The proportion of individuals shedding on the day of and 4 and 7 days after the 6-week dose of RV1. Shedding at up to 4.2 million copies per reaction was observed in post-vaccination samples. Abbreviation: RV, rotavirus.

OTU ID	Taxonomy
14490	*Bifidobacterium adolescentis*
22099	*Escherichia coli*
6490	*Streptococcus*
17699	Enterobacteriaceae sp.
20859	*Streptococcus*
866	*Lactobacillus*
15701	*Bacteroides fragilis*
24098	Enterococcaceae sp.
5022	*Rothia mucilaginosa*
5609	*Veillonella*
5371	*Collinsella*
20653	*Bifidobacterium*
18905	*Streptococcus luteciae*
21300	*Lactobacillus zeae*
14371	*Staphylococcus*
12381	*Lactobacillus*
21986	*Veillonella dispar*
18372	*Clostridium*
20987	*Bacteroides caccae*
16006	*Bacteroides*

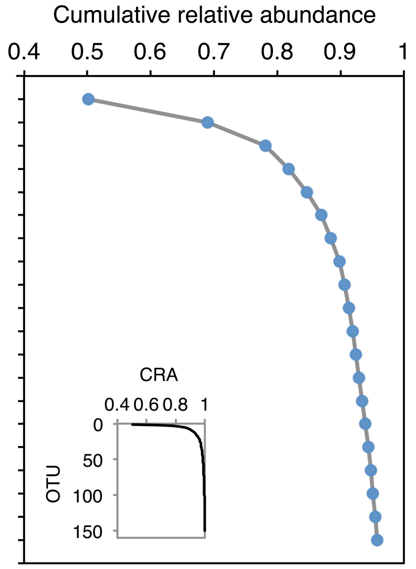

Supplementary Figure 3. Top 20 OTUs at 6 weeks of age. The abundance distribution of all OTUs is displayed in the inset. When ranked by decreasing mean relative abundance, 18 OTUs accounted for >95% of the observed microbiota in these samples. Abbreviations: CRA, cumulative relative abundance; OTU, 97%-identity operational taxonomic unit; w, weeks.

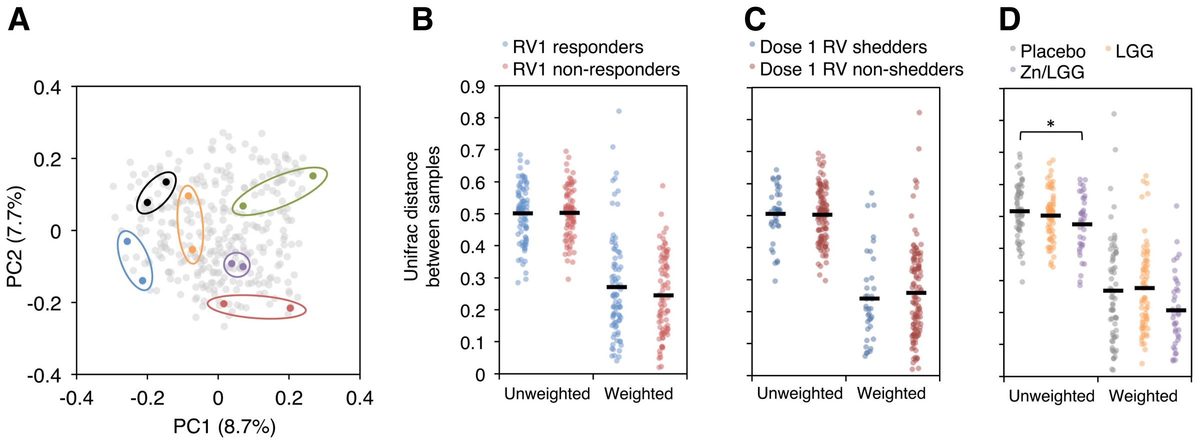

Supplementary Figure 4. Association between microbiota stability and Rotarix response. (A) Clustering of 6- and 10-week samples within each individual based on unweighted Unifrac distances. Six sample pairs are highlighted. (B, C, D) Within-subject Unifrac distances, used as an indicator of microbiota stability, are displayed according to (B) seroconversion status, (C) RV1 take, and (D) study arm. Mean values in each group are indicated. * $P < 0.05$. Abbreviations: PC, principal coordinate; RV, rotavirus; RV1, Rotarix.

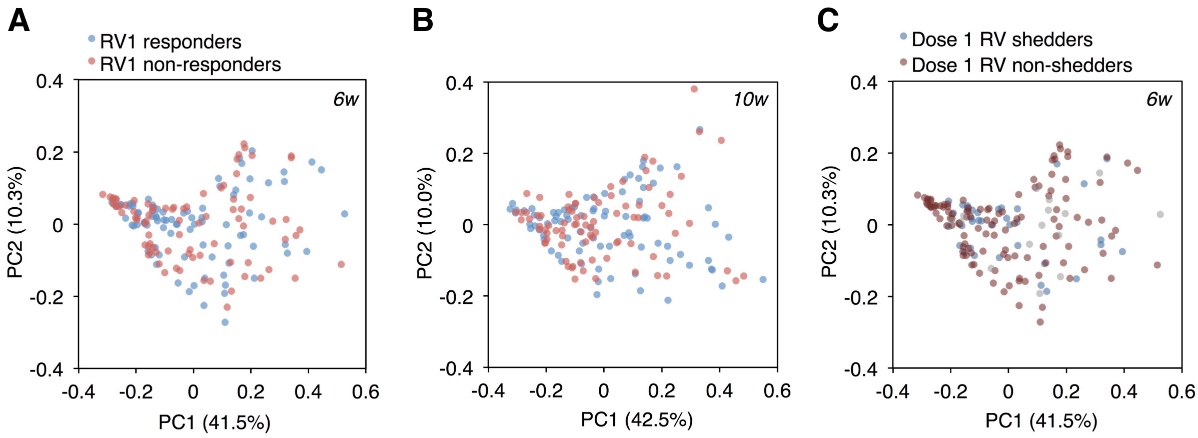

Supplementary Figure 5. Clustering of samples according to Rotarix response. Weighted Unifrac distances between samples, visualized via principal coordinates analysis, are displayed at (A) 6 weeks and (B) 10 weeks according to seroconversion status, and (C) according to vaccine take following the 6-week RV1 dose. Abbreviations: PC, principal coordinate; RV, rotavirus; RV1, Rotarix.

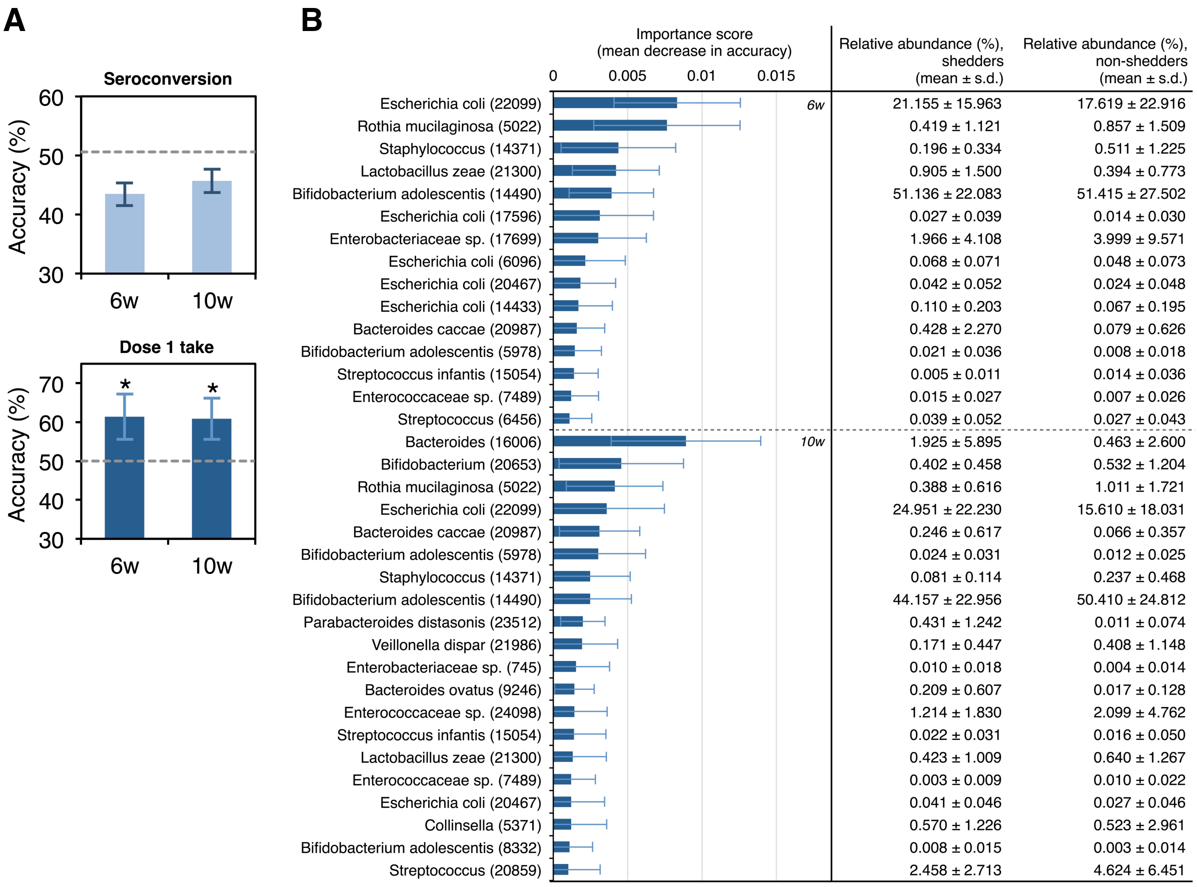

Supplementary Figure 6. Predictive accuracy of Random Forest models for Rotarix outcome. Mean accuracy (± standard deviation) across 100 iterations of the Random Forest algorithm is displayed for models predicting seroconversion status (upper) and dose 1 RV1 take (lower), where accuracy = 100 – out-of-bag error rate. Dotted lines indicate baseline accuracy, wherein all individuals are assigned to the majority class. Separate analyses were carried out for 6- and 10-week samples. (B) Highest ranking taxa (and corresponding OTU IDs) by Random Forest importance score (mean ± standard deviation) for prediction of dose 1 RV1 take. * P <0.05. Abbreviations: s.d., standard deviation; w, weeks.

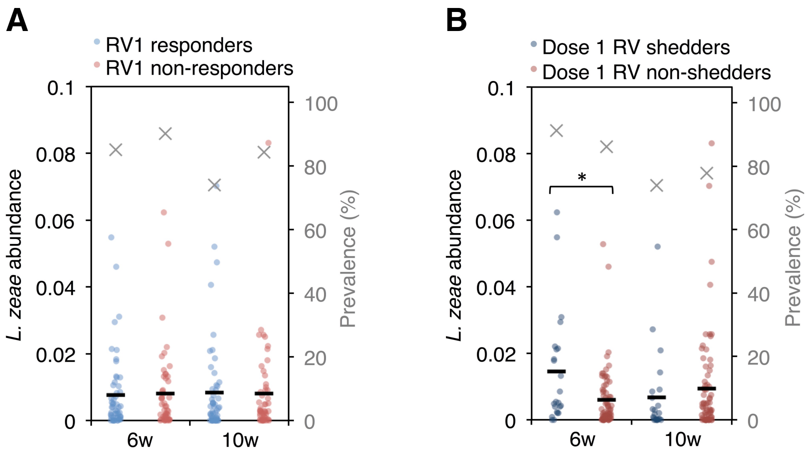

Supplementary Figure 7. Association between probiotic strain abundance and Rotarix response. (A) Among probiotic recipients (either with or without zinc), relative abundance of the enriched *Lactobacillus* strain (OTU 21300) is displayed according to (A) seroconversion status (non-parametric *t* test, *P* values of 0.835 and 0.897 for 6- and 10-week comparisons, respectively; n = 105, of whom 54 were responders) and (B) dose 1 RV1 take (non-parametric *t* test, *P* values of 0.014 and 0.490 for 6- and 10-week comparisons, respectively; n = 95, of whom 23 were shedders). Mean relative abundance is indicated by a horizontal line and prevalence by a cross. * $P < 0.05$ (no FDR correction).

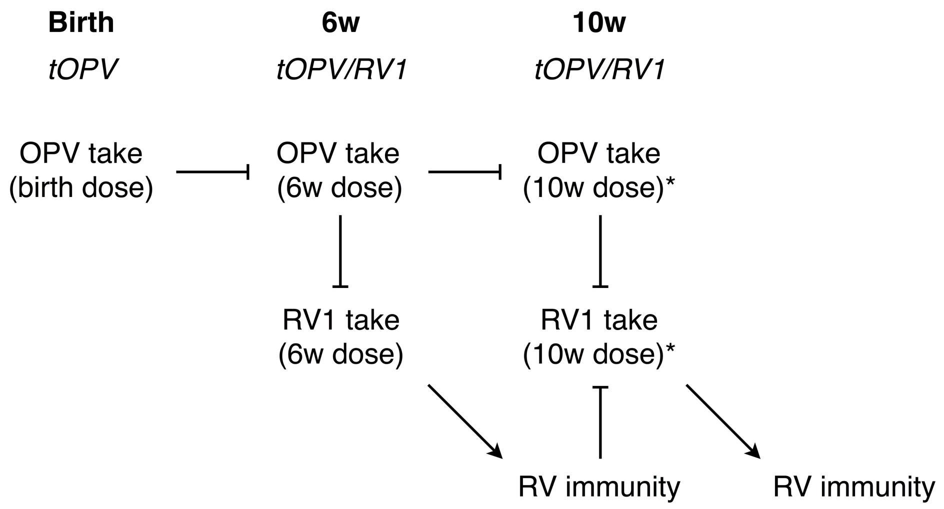

Supplementary Figure 8. Conceptual model of interactions between oral rotavirus and poliovirus vaccines administered in early infancy. * outcome not measured in this study. Abbreviations: OPV, oral poliovirus vaccine; RV, rotavirus; RV1, Rotarix; tOPV, trivalent oral poliovirus vaccine; w, weeks.

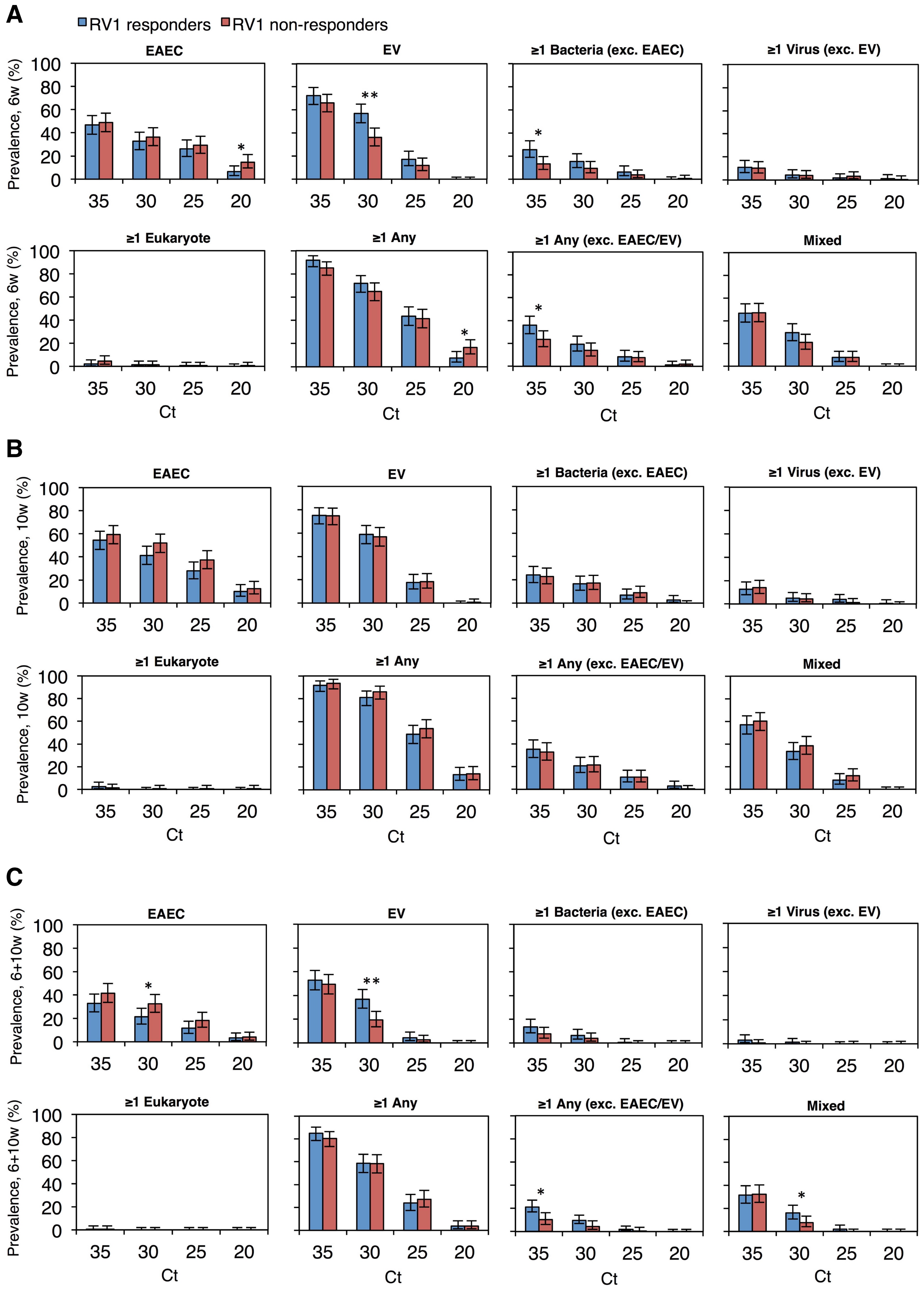

Supplementary Figure 9. Impact of threshold cycle on the association between concurrent pathogens and Rotarix immunogenicity. The proportion of infants infected with the specified pathogen or pathogen group at (A) 6 weeks, (B) 10 weeks, and (C) both timepoints is displayed. Rotavirus infections were excluded. * *P* <0.05; ** *P* <0.005. Abbreviations: Ct, threshold cycle; EAEC, enteroaggregative *Escherichia coli*; EV, enterovirus; RV1, Rotarix.

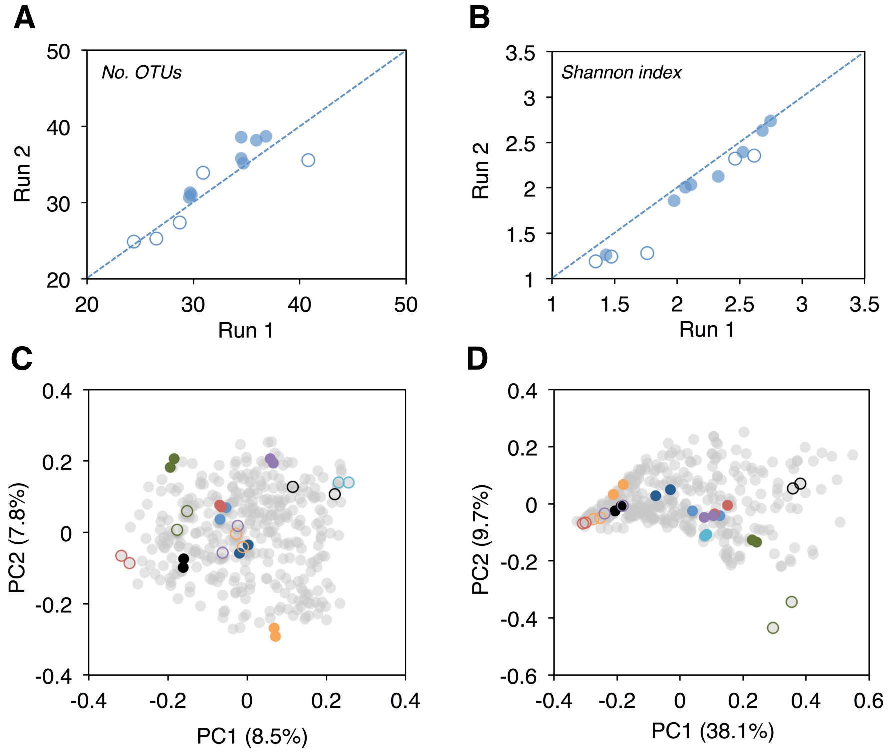

Supplementary Figure 10. Comparison of technical replicates. Concordance between technical replicates is shown for (A) number of OTUs, (B) Shannon index, (C) unweighted Unifrac distances, and (D) weighted Unifrac distances. The same PCR product was re-sequenced in eight of these samples (filled circles), while PCR was repeated for the remaining five (non-filled circles). Abbreviations: OTU, 97%-identity operational taxonomic unit; PC, principal coordinate.
